# Pteropods make thinner shells in the upwelling region of the California Current Ecosystem

**DOI:** 10.1038/s41598-021-81131-9

**Published:** 2021-01-18

**Authors:** Lisette Mekkes, Willem Renema, Nina Bednaršek, Simone R. Alin, Richard A. Feely, Jef Huisman, Peter Roessingh, Katja T. C. A. Peijnenburg

**Affiliations:** 1 Naturalis Biodiversity Center, Leiden, The Netherlands; 2 Institute for Biodiversity and Ecosystem Dynamics, University of Amsterdam, Amsterdam, The Netherlands; 3 Southern California Coastal Water Research Project, Costa Mesa, CA USA; 4 National Institute of Biology, Ljubljana, 1000 Slovenia; 5 Pacific Marine Environmental Laboratory, National Oceanic and Atmospheric Administration, Seattle, WA USA

**Keywords:** Marine biology, Climate change, Ecology, Biogeochemistry

## Abstract

Shelled pteropods are widely regarded as bioindicators for ocean acidification, because their fragile aragonite shells are susceptible to increasing ocean acidity. While short-term incubations have demonstrated that pteropod calcification is negatively impacted by ocean acidification, we know little about net calcification in response to varying ocean conditions in natural populations. Here, we examine in situ calcification of *Limacina helicina* pteropods collected from the California Current Ecosystem, a coastal upwelling system with strong spatial gradients in ocean carbonate chemistry, dissolved oxygen and temperature. Depth-averaged pH ranged from 8.03 in warmer offshore waters to 7.77 in cold CO_2_-rich waters nearshore. Based on high-resolution micro-CT technology, we showed that shell thickness declined by ~ 37% along the upwelling gradient from offshore to nearshore water. Dissolution marks covered only ~ 2% of the shell surface area and were not associated with the observed variation in shell thickness. We thus infer that pteropods make thinner shells where upwelling brings more acidified and colder waters to the surface. Probably the thinner shells do not result from enhanced dissolution, but are due to a decline in calcification. Reduced calcification of pteropods is likely to have major ecological and biogeochemical implications for the cycling of calcium carbonate in the oceans.

## Introduction

Since the start of the industrial revolution, the oceans have absorbed about one-quarter of the anthropogenic carbon dioxide emission^1,2^. As a result, surface ocean pH has decreased by ~ 0.1 globally, a process referred to as ‘anthropogenic ocean acidification’^3–5^. Ocean acidification is accompanied by lower carbonate ion concentrations, and decreasing saturation states of aragonite and calcite. This will have important consequences for a wide variety of marine organisms, particularly those that produce carbonate shells or skeletons, such as coccolithophores, foraminifers, molluscs, and corals^6–12^. Shelled pteropods, a group of holoplanktonic gastropods, represent sentinel organisms to assess the impact of ocean acidification because of their thin aragonite shells^13–16^.

Incubation experiments with pteropods have revealed that acidified conditions are associated with enhanced shell dissolution^13^, higher mortality^17^, and reduced calcification rates^16–18^. Significant changes have also been found in expression of genes involved in neurofunction, ion transport, and shell formation^19–21^. Field data demonstrate that changes in current ocean carbonate chemistry already impact natural populations of shelled pteropods^13,14,17,22,23^. While there is an indication that pteropods can counter dissolution due to the protection provided by an organic layer, the periostracum, and short-term repair capacity^24,25^, the extent to which these mechanisms offset the sensitivity of pteropods to ocean acidification is currently unknown^24,25^. Understanding variability in pteropod net calcification along natural gradients is important to give insight into their sensitivity and potential ability to cope with a changing environment.

Pteropods require a positive balance between precipitation and dissolution processes to form a shell. This balance will determine basic shell characteristics, such as growth and thickness, and depends on the environmental conditions pteropods experience during their lifetime^19^. While shell dissolution has been proposed as an indicator of the effects of anthropogenic ocean acidification^14^, shell thickness reflects the net effect of shell formation and dissolution. For instance, comparison of historical (from 1921) and modern samples of the pteropod *Styliola subula* demonstrated that shells had become thinner with a decrease in pH in the Mediteranean Sea^26^. Shell thickness has thus been proposed as a useful indicator of the effect of environmental change across various temporal scales^26,27^.

The California Current Ecosystem (CCE) experiences acidified conditions due to a combination of natural and anthropogenic factors^22,28^. Seasonal upwelling brings up cold and aragonite undersaturated waters (Ω_Ar_ < 1) with high *p*CO_2_ and low pH from the offshore intermediate waters of the northern Pacific onto the continental shelf^22,28,29^. In addition, the exchange and downward mixing of anthropogenic CO_2_ from global and local atmospheric sources^4,22,30^, and increased respiration at intermediate and bottom depths leads to further shoaling of these aragonite-undersaturated waters^28^. In particular, the northern CCE region shows locally low pH levels throughout the coastal region. The CCE thus represents strong environmental onshore-offshore gradients due to upwelling of advected cold and acidified water in combination with anthropogenic ocean acidification and with respiration processes occurring in the water column.

Shells of *Limacina helicina* pteropods were reported to show signs of dissolution in the upwelling waters of the CCE, where Ω_Ar_ values near or below undersaturation were observed^14,22,23^. Moreover, calcein-staining of pteropods shells has indicated reductions in gross calcification related to these acidified conditions^17^. Yet, better quantification of the effects of corrosive conditions on net calcification in natural populations of shelled pteropods will be needed, the understanding of which carries both ecological and biogeochemical importance.

Here, we examine the variability in net calcification of *L. helicina* pteropods along the upwelling gradients of the CCE, characterized by a combination of parameters, including low pH and Ω_Ar_, low temperature, low oxygen and high *p*CO_2_. We measured shell thickness of 80 individuals as a proxy of net calcification using micron-scale computed tomography (micro-CT), a high-resolution X-ray technique that provides detailed 3D information on the shell^25–27^. In addition, the extent of dissolution on the outer surface of the same shells was examined through Scanning Electron Microscopy (SEM) to assess to what extent dissolution level impacts shell thickness. We also verified that all examined individuals belong to a single population of *Limacina helicina* based on partial sequences of the commonly used DNA barcoding gene Cytochrome Oxidase I gene (COI). This study quantifies pteropod calcification in natural populations and indicates that not shell dissolution, but reduced calcification is the primary process impacted along upwelling gradients in the CCE.

## Results

### Ocean carbonate chemistry

**Figure 1:**
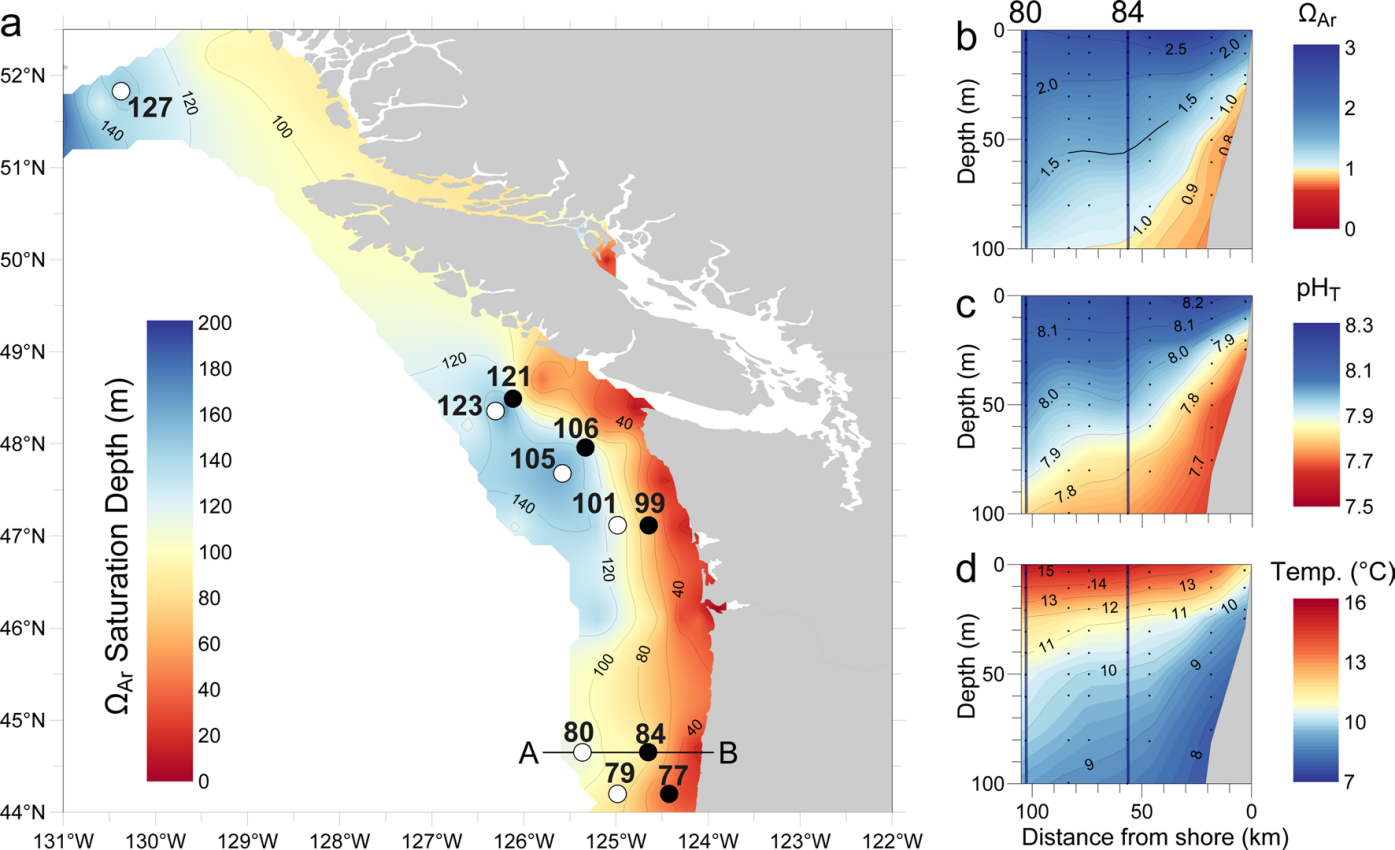
Aragonite saturation along the northern California Current Ecosystem. (**a**) Map representing the aragonite saturation horizon, defined as the depth at which Ω_Ar_ = 1, between May 28th and June 7th of 2016. Waters with Ω_Ar_ < 1 are considered undersaturated. *Limacina helicina* pteropods were sampled from 11 stations, indicated by open and closed symbols for offshore and nearshore stations, respectively. (**b–d**) Depth distributions of (**b**) Ω_Ar_, (**c**) pH_T_, and (**d**) temperature (°C), along the offshore-onshore gradient from locations A to B indicated on the map. Each dot in (**b**–**d**) indicates a sampling point, and stations 80 and 84 are indicated by vertical lines. Undersaturated conditions (Ω_Ar_ < 1) come close to the surface in nearshore waters and deepen offshore.

**Figure 2:**
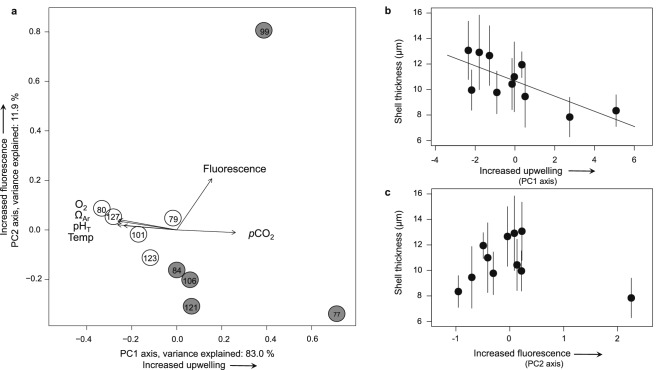
Relationships among ocean variables and average shell thickness of *L. helicina* pteropods. (**a**) Principal Component Analysis (PCA) based on aragonite saturation (Ω_Ar_), *p*CO_2_, pH, temperature (Temp), oxygen (O_2_) and chlorophyll fluorescence. The numbers within the plot represent the sampling stations used for analyses. Circles indicate stations with strong upwelling conditions nearshore (grey), and with less intense upwelling conditions offshore (white), see also Fig. 1. PC1 explains 83.0% of the variation among stations, and essentially reflects the offshore-onshore gradient driven by coastal upwelling. PC2 explains 11.9% of the variation, and is largely driven by variation in chlorophyll fluorescence. (**b**) Average shell thickness decreased significantly with PC1, associated with the upwelling gradient (principal component regression: y =  − 0.595 PC1 + 10.671; R^2^ = 0.487, N = 11, p = 0.010). (**c**) Average shell thickness did not vary significantly with PC2, associated with chlorophyll fluorescence (principal component regression: R^2^ =  − 0.058, N = 11, p = 0.519). Each dot in (**b**,**c**) represents the mean ± SD of average shell thickness calculated over all 6–8 individuals per station. PC axes in the biplot (**a**) were scaled according to Gabriel (1971), whereas PC axes in (**b**,**c**) are unscaled.

In May–June 2016, coastal upwelling of cold and relatively CO_2_-rich waters caused strong spatial variation in Ω_Ar_, pH and temperature (Fig. 1). The aragonite saturation horizon (defined as the depth at which Ω_Ar_ = 1) was relatively close to the surface (at 20–40 m depth) near the coast, but much deeper (below 50 and 130 m) in the offshore regions (Fig. 1a) due to coastal upwelling. This resulted in a pronounced acidification gradient, with a depth-averaged Ω_Ar_ over the upper 100 m of the water column ranging from 1.08 onshore to 1.79 offshore and depth-averaged pH ranging from 7.77 to 8.03 (Fig. 1b,c, Table 1). In addition, depth-averaged temperature and dissolved oxygen concentrations also increased substantially from the onshore to the offshore stations (Fig. 1d, Table 1). In fact, all measured ocean variables (Ω_Ar_, pH, *p*CO_2_, temperature, oxygen) except chlorophyll fluorescence, were strongly correlated with each other along this upwelling gradient (Fig. 2a). PC1 explains 83.0% of the environmental variability among stations, and essentially reflects the offshore-onshore gradients driven by coastal upwelling. PC2 explains 11.9% of the variability, and is largely related to variation in chlorophyll fluorescence (Fig. 2a).

**Table 1:** Oceanographic variables measured at the eleven stations in the California Current Ecosystem from which the pteropods were sampled.

Station	Ω_Ar_	pH	O_2_ (µmol/kg)	Temp. (°C)	Chlorophyll fluorescence	*p*CO_2_ (ppm)
77	1.08	7.77	154.70	8.84	0.09	828.01
79	1.57	7.94	226.70	10.35	0.07	526.21
80	1.79	8.01	249.80	11.16	0.04	429.29
84	1.57	7.93	218.60	10.35	0.04	542.96
99	1.39	7.87	207.50	9.31	0.24	653.04
101	1.71	7.98	240.60	10.44	0.04	471.52
105	1.73	7.99	247.60	10.77	0.04	439.55
106	1.50	7.93	226.00	9.63	0.04	521.33
121	1.48	7.93	223.90	9.43	0.03	525.33
123	1.62	7.97	244.50	10.03	0.03	465.39
127	1.74	8.03	264.60	10.25	0.04	394.08

Values were averaged over the upper 100 m of the water column. The locations of the stations are shown in Fig. 1a.

Aragonite saturation (Ω_Ar_), oxygen (O_2_), temperature (Temp).

### Shell thickness

**Figure 3:**
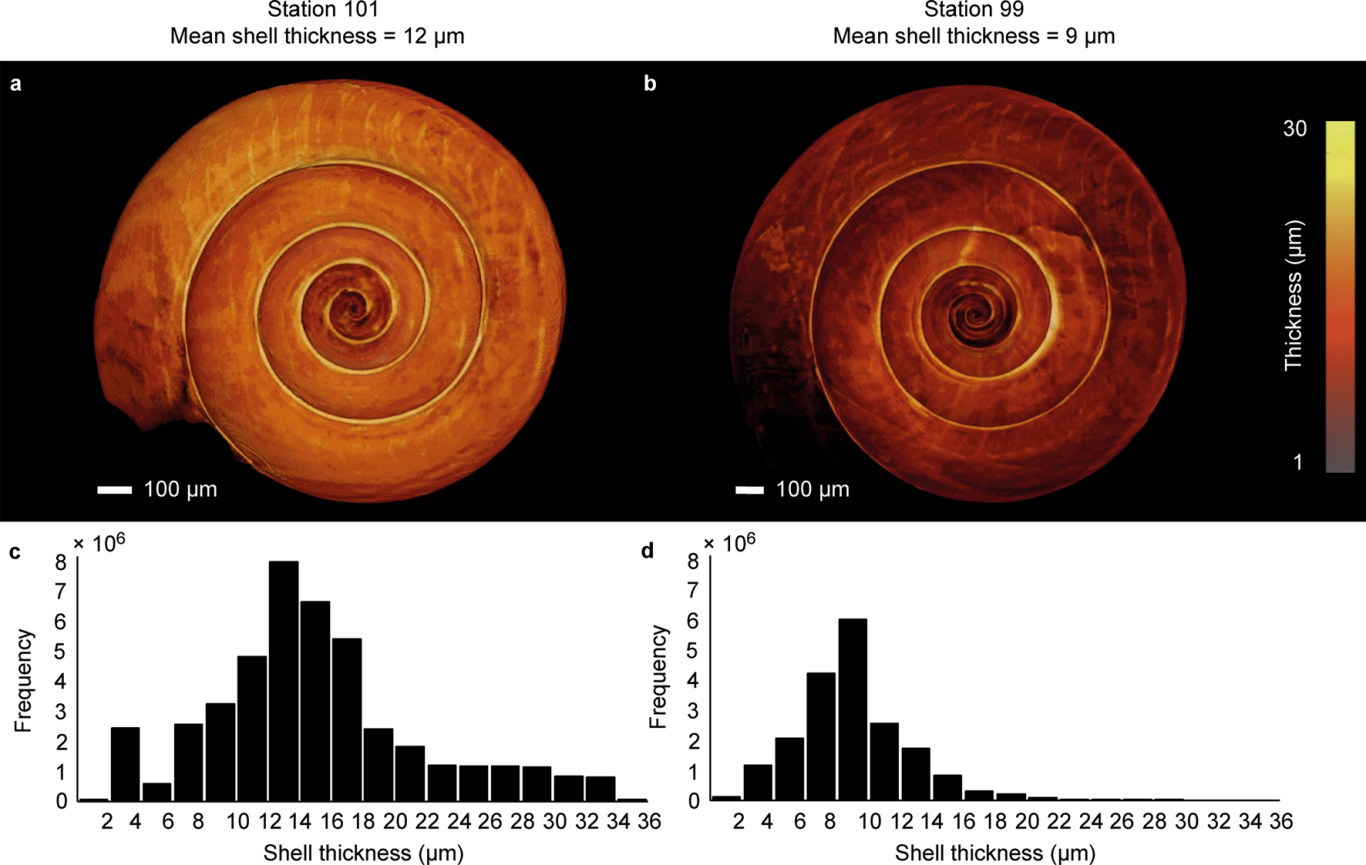
Variation in shell thickness measured by micro-CT scans of two *Limacina helicina* specimens. (**a**) A thick shell sampled offshore (station 101). (**b**) A thin shell sampled onshore (station 99). Coloring indicates shell thickness, with brighter colors for thicker areas. (**c**,**d**) Frequency distribution of shell thickness of the two specimens. These frequency distributions were used to calculate average thickness per shell.

**Figure 4:**
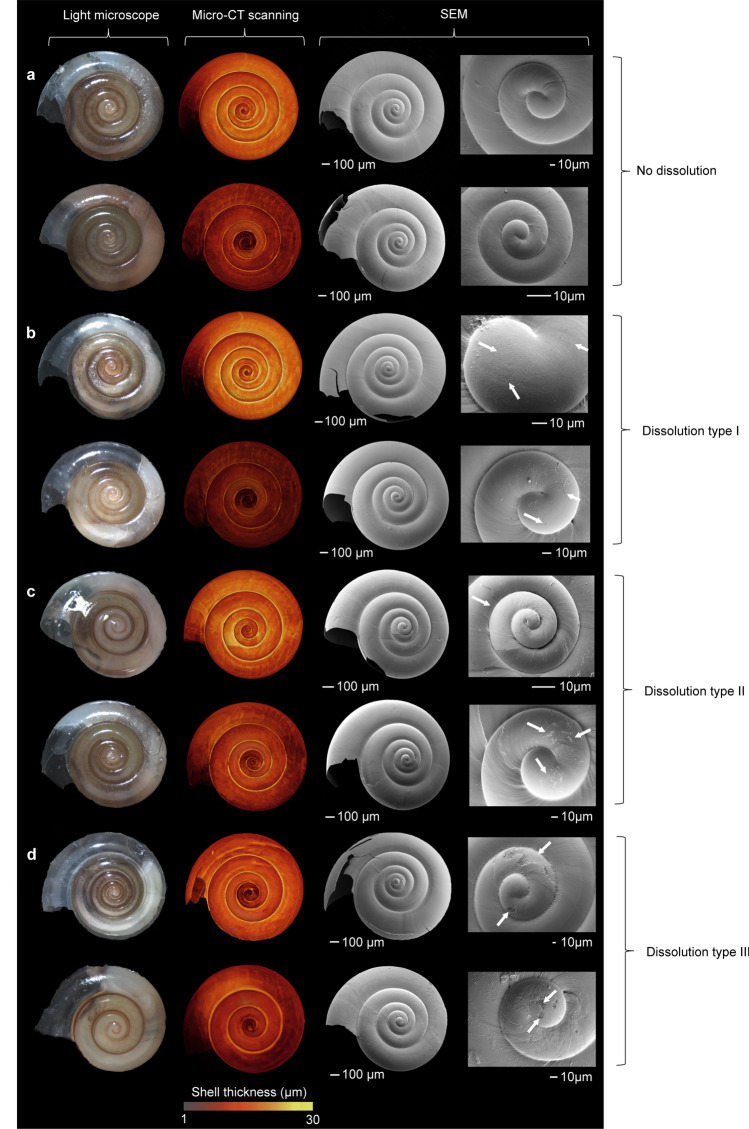
Shell images obtained by light microscopy, micro-CT and scanning electron microscopy (SEM) of eight *Limacina helicina* specimens illustrating the variability in our data set. (**a**) Specimens with a thick shell (upper row) and a thin shell (lower row) without dissolution marks. (**b**) Specimens with a thick shell and a thin shell showing marks of initial dissolution (Type I), with pitting at the inner whorl (indicated by arrows). (**c**) Specimens with a thick shell and a thin shell with Type II marks of dissolution penetrating the aragonite structure. (**d**) Specimens with a thick shell and a thin shell with Type III dissolution, characterized by deep damage on the outer shell surface. All images in the same row are of the same specimen.

Shell diameter, height and thickness were measured based on 3D models acquired by micro-CT scanning of 80 *Limacina helicina* individuals (Fig. 3, Tables S1, S2). Analyzed specimens had a shell diameter ranging from 0.9 to 3.0 mm (Fig. S1), which, according to the conceptual life cycle model of Wang et al.^31^, means that they are most likely from the spring generation. Because a recent study using particle tracking demonstrated sustained retention for 5–6 weeks in the coastal regions of the CCE during the upwelling season^17^, we considered the in situ oceanographic variables measured at the time and place of pteropod collection to be representative of the conditions they experienced during most of their lifetime. All shells had a thin initial whorl compared to the rest of the shell, and a localized thickening at the base of the shell towards the aperture (Figs. 3, 4). The frequency distributions of shell thickness had a similar shape for all shells (Fig. S2), indicating that shell thickening or thinning occurs more or less evenly across the entire shell. We calculated the average thickness per shell from these frequency distributions. We found that average shell thickness was not correlated with shell diameter, shell height, or number of whorls (Pearson’s product-moment correlation: r = 0.13, 0.12, 0.11, respectively, p > 0.2; Fig. S3a–c). Hence, average shell thickness of *L. helicina* was unrelated to the age or size of the individuals.

Our results show that average shell thickness declined significantly along the upwelling gradient (PC1 axis), with thinner shells at nearshore stations with stronger upwelling conditions (Fig. 2b; principal component regression: R^2^ = 0.487, N = 11, p = 0.010). Average shell thickness did not vary significantly with the PC2 axis associated with chlorophyll fluorescence, a proxy for food availability (Fig. 2c; principal component regression: R^2^ =  − 0.058, N = 11, p = 0.519). Average shell thickness varied significantly among stations (Fig. S4; one**-**way ANOVA: F_10,69_ = 6.064, p < 0.001), and individuals from nearshore stations had thinner shells (7.84 ± 1.56 µm (mean ± SD), N = 37) than individuals from offshore stations (13.07 ± 2.29 µm, N = 43). Figure 3 provides two examples. Based on the principal component regression of average shell thickness and PC1 (Fig. 2b) we calculate that shell thickness declined by ~ 37% along the offshore-onshore gradients generated by coastal upwelling in the CCE.

### Shell dissolution

**Figure 5:**
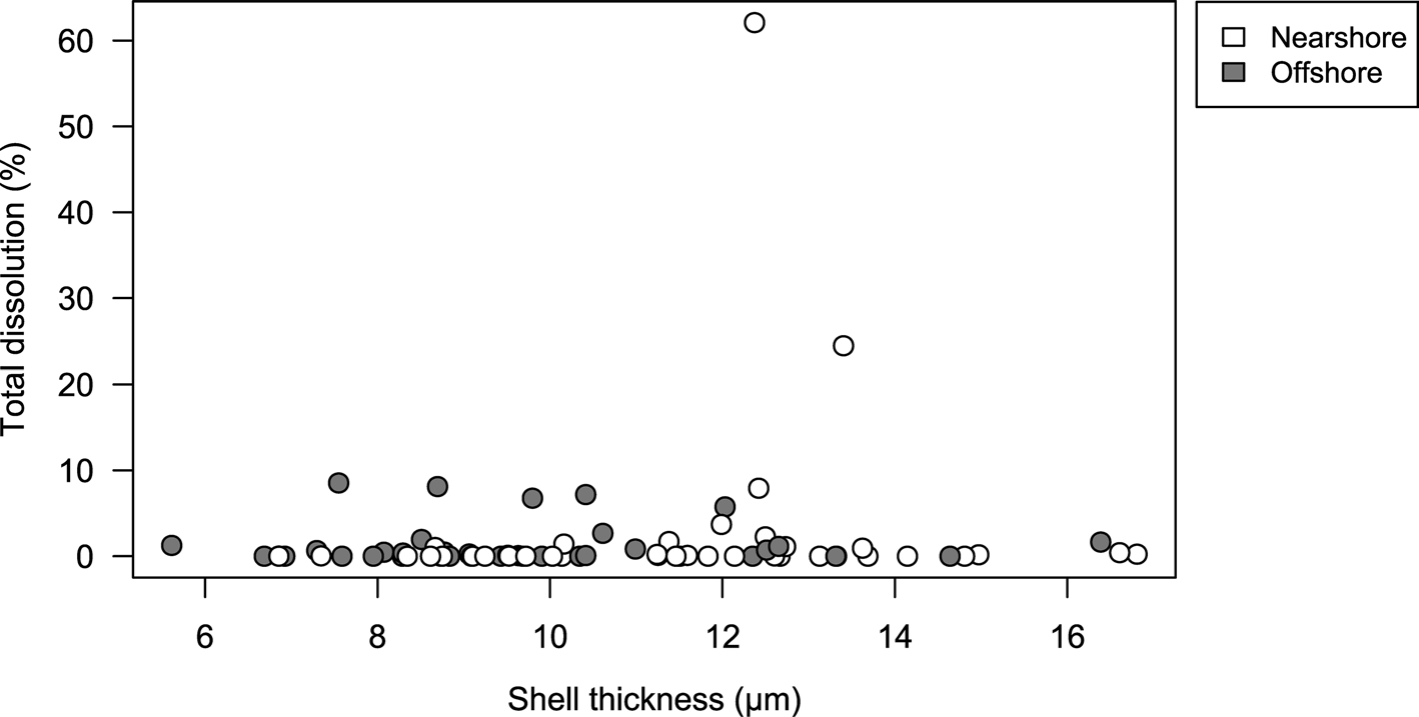
Percentage surface area with dissolution marks (%) and average shell thickness of the individual shells. Each dot represents an individual pteropod shell analyzed for both average shell thickness and dissolution marks. Circles indicate individuals from nearshore stations with strong upwelling conditions (grey), and offshore stations with less intense upwelling conditions (white). See Fig. 4 for examples of dissolution marks, Fig. S5 for specific graphs of the three different dissolution types, and Fig. S8 for an illustration of the calculation method.

We inspected the shell surface for dissolution marks using SEM on a subset of 76 individuals with unbroken shells after micro-CT scanning. We used the dissolution types as defined by Bednaršek et al.^14^, including initial dissolution (Type I; Fig. 4b) and more severe types of dissolution (Type II and Type III; Fig. 4c,d) and expressed dissolution as percentage of the shell surface area showing dissolution marks. We found dissolution marks only on the inner two whorls (Fig. 4b–d), where the marks covered on average 2.1 ± 0.9% (mean ± SE, N = 76) of the surface area. The inner whorls are the oldest part of the shell and, hence, exposed to the external environment the longest. The percentage surface area affected by dissolution was not correlated with average shell thickness (Pearson’s product-moment correlation: r = 0.112, p = 0.334; Fig. 5), irrespective of the type of dissolution (Fig. S5).

### Population structure

Analysis of the CO1 sequence data confirmed that all examined individuals belong to a genetically homogeneous population of *Limacina helicina* (non-significant genetic differentiation among stations: Φst < 0.045, p > 0.4). This population is characterized by relatively low levels of genetic diversity (nucleotide diversity (π) of 0.0026 ± 0.0018; Fig. S6a) compared to populations of the same or related species. The CCE population was not genetically distinct from *L. helicina* populations in the western North Pacific (0.07% average sequence divergence)^32^, and separated by 0.46% and 34.6% average sequence divergence from *L. helicina* populations in the Arctic and Antarctic, respectively^33,34^ (Fig. S6b).

## Discussion

Based on a combination of micro-CT scans, SEM images, and in situ ocean chemistry observations, we found that pteropods produce thinner shells in coastal waters of the CCE than further offshore. More specifically, our results show that intense upwelling in these coastal areas, characterized by CO_2_-rich waters with low pH and Ω_Ar_ as well as low temperature and dissolved oxygen concentrations, was associated with a substantial decline in shell thickness (Fig. 2b). Dissolution marks were not widespread on pteropod shells and shell thickness was not correlated with the level of dissolution (Figs. 5, S5). Therefore, the decline in shell thickness was probably not the result of enhanced dissolution, but rather the consequence of reduced calcification in response to environmental conditions associated with the upwelling gradients. Ocean carbonate chemistry, dissolved oxygen and temperature strongly covary in the CCE (Fig. 2a); hence, a single “best predictor” cannot be selected to explain differences in shell thickness based on these field observations only. The different ocean variables could independently or concurrently intensify or counterbalance the environmental impact on pteropod calcification^23,35^. Here, we discuss the potential impacts of ocean carbonate chemistry, oxygen and temperature on pteropod calcification to further unravel their contributions to our field observations in the CCE.

First, spatial variation in ocean carbonate chemistry provides a plausible explanation for the observed variation in shell thickness of *L. helicina* pteropods. Thin shells were found in coastal waters of the CCE with high *p*CO_2_, low pH and aragonite saturation levels approaching undersaturated conditions (Ω_Ar_ = 1.08), whereas thick shells were found in offshore stations that were supersaturated with aragonite (Ω_Ar_ up to 1.79). Pteropods are likely to produce less shell material if the major substrate (carbonate) required for shell formation is in short supply. This is in agreement with numerous experimental studies, showing that acidified conditions reduce calcification^16,18–20^, as well as in situ measurements using calcein-stained shells^17^, and a synthesis study showing that pteropod calcification was negatively affected when Ω_Ar_ is below 1.2^35^. The 37% decline in shell thickness along the upwelling gradient of our study exceeds the 25% decline in shell thickness over the past ~ 100 years reported for *Styliola subula* from the Mediterranean Sea, where waters are still super-saturated with respect to aragonite^26^.

Second, although oxygen is one of the ocean variables strongly associated with the upwelling gradient (Fig. 2a), it is unlikely to have impacted pteropod calcification. Surface waters at all stations were well oxygenated, with oxygen concentrations never below 150 µmol kg^−1^ (Table 1), which is well above values where mollusks could be physiologically compromised^36^. The observed offshore–onshore variability in oxygen concentrations likely reflects upwelling of deep waters with relatively low oxygen contents as a result of respiratory processes in the deep.

Third, temperature was strongly associated with the upwelling gradient (Fig. 2a) and is likely to have an impact on calcification. We found that *L. helicina* produced thinner shells nearshore, where waters were relatively cold and more acidified compared to offshore (Table 1). Temperature is known to have a strong influence on the shell-building capacity of calcifying organisms with several studies showing that increasing temperatures stimulate shell growth (reviewed by Gazeau et al.^8^). In the CCE, temperature varies strongly among seasons and across vertical and horizontal gradients^37^. For example, in our study, depth-averaged water temperature over the upper 100 m ranged from 8.84 °C nearshore to 11.16 °C offshore (Table 1). It is therefore possible that warmer waters offshore enhanced calcification. Hence, both temperature and ocean carbonate chemistry may have contributed to the observed variation in shell thickness along the upwelling gradient of the CCE. Future studies will be needed to further disentangle the relative contribution of these important environmental variables.

Fourth, we did not find a significant relationship among shell thickness and the PC2 axis, reflecting chlorophyll fluorescence, a proxy for food availability (Fig. 2c). Notably, we collected the thinnest shells at the onshore station with the highest chlorophyll fluorescence (station 99). This is in contrast to previous studies, which reported that increased food availability enhanced calcification in bivalves^38^ and pteropods^27,39,40^. During spring, at the onset of the upwelling season, phytoplankton blooms in the productive waters of the CCE are commonly found close to shore and are often patchy^41^. It is still possible that ample food in the CCE helped pteropods to sustain growth and calcification, but our results indicate that the ocean parameters associated with PC1 are more likely to be primary drivers of calcification.

Although *L. helicina* pteropods produce thinner shells along the upwelling gradient in the CCE, we did not find increased dissolution of the shells along the upwelling gradient (Fig. 5). This contrasts with previous findings in the same region^14,42^, which reported more severe dissolution marks of *L. helicina* and found that shell dissolution was correlated with decreased Ω_Ar_ conditions. In our study, pteropods were collected at the start of the upwelling season in May, and, therefore, experienced shorter exposure to corrosive conditions compared to these previous studies, which collected *L. helicina* later in the upwelling season in August^14,42^. It is thus possible that more severe shell dissolution was observed in previous studies due to a longer exposure to corrosive conditions.

The ecological implications and potential adaptive significance of variation in shell thickness are as yet unknown for pteropods. While pteropod shells protect against predation and infections^43^, making thinner shells could also be an adaptive or acclimation strategy. Thinner shells were not related to a smaller shell size (Fig. S3a–c), and shell size was not related to ocean parameters, suggesting that growth of the *L. helicina* individuals was unaffected along the upwelling gradients. An increase in size is necessary to reach fertility and produce offspring^44^ and is thus likely under evolutionary pressure. The production of thinner shells in colder and more acidified waters close to shore could therefore be the result of an acclimative response by reallocating energy to sustain growth while reducing the energetically expensive process of calcification. Little is known about the formation of pteropod shells, but it is likely under strong biological control, as found for other mollusks^45,46^. To obtain a more comprehensive understanding of the underlying mechanisms by which pteropods produce thinner shells in stronger upwelling conditions, future efforts could focus on measuring the interplay between different physical–chemical and biological processes involved in calcification, e.g. by assessing differential gene expression in onshore and offshore populations of *L. helicina*, or by careful analysis of potential interactions between covarying stressors using experimental manipulations (e.g.,^10,47^).

Naturally upwelled high-CO_2_ water combined with a rapid increase of atmospheric CO_2_ concentrations make coastal upwelling areas like the CCE particularly susceptible to ocean acidification processes^22,30^. Already in the year 2050, more than half of the waters in the CCE will be aragonite-undersaturated year-round^28,48^. Globally, pteropods contribute at least 33% to shallow (100 m) export of CaCO_3_ out of surface waters^49^. Hence, the consequence of further acidification, resulting in reduced aragonite shell-production of pteropods, may have major implications for CaCO_3_ export flux^49^. Although making thinner shells could be an energetically competitive strategy, the question remains for how long pteropods can continue making thinner shells to sustain themselves in increasingly acidified waters.

## Methods

### Specimen and ocean data collection

*Limacina helicina* specimens were collected from 44 to 51° N and 124 to 130° W in the California Current Ecosystem (see Fig. 1a) on board NOAA Ship *Ronald H. Brown* during the spring of 2016 (May 27th to June 5th). Each transect was conducted in a perpendicular orientation to the coastline to cross offshore–onshore oceanographic gradients driven by upwelling. Collection methods were described by Bednaršek et al.^14^. *Limacina helicina* specimens were encountered on six transects, from Oregon (USA) to British Columbia (Canada). Stations for analyses were selected based on their location; either furthest or closest to shore, in order to span the full upwelling gradient. Actively moving, undamaged specimens were selected from the net tows and fixed in 96% ethanol for micro-CT scanning, SEM imaging, and DNA barcoding. The ethanol was replaced after 24 h and samples were maintained at − 20 °C. In total, 238 *L. helicina* specimens were obtained for this study (Table S1). At each of the stations, oceanographic variables were sampled at the same time as the specimens were collected to characterize their habitat^50^. This included CTD (conductivity-temperature-depth) casts equipped with an auxiliary Chlorophyll Fluorometer (Seapoint Sensors, Inc., Exeter, NH, USA) to measure vertical profiles of temperature (T), salinity (S), and fluorescence. At each station, water samples were collected at different depths using modified Niskin-type bottles and analyzed onboard the ship for dissolved inorganic carbon (DIC), total alkalinity (TA), and oxygen using the methods described in Feely et al.^22^. The DIC and TA samples were poisoned with HgCl_2._ Phosphate, silicate, and other nutrient concentrations were analyzed after the cruise according to Alin et al.^29^. From these data (i.e., DIC, TA, Temperature, Salinity, pressure, and phosphate and silicate concentrations (Table S3), we calculated pH_T_, *p*CO_2_, and Ω_Ar_, using Lueker et al. (2000) dissociation constants^51^. The oceanographic variables were integrated over the upper 100 m of the water column to obtain mean values (Table 1), because most *L. helicina* individuals in the coastal waters of the NE Pacific remain in the upper 100 m during day and night^50^.

### Pteropod shell analyses

*Limacina helicina* specimens (Tables S1, S2) were imaged with a stacking microscope (Zeiss SteREO Discovery V20), and subsequently scanned using a micro-CT scanner (SkyScan, model 1172, Aartselaar, Belgium). Because micro-CT scanning and 3D reconstructions are time-consuming, we selected eight specimens per station based on the best preserved shells (i.e. shells without large cracks induced by mechanical damage through collection and handling). A few shells moved during CT-scanning and these data were discarded, resulting in micro-CT scans for 80 specimens in total. Specimens were carefully placed in pipet tips and mounted in stacks of maximum three individuals for micro-CT scanning. This method provided protection of the fragile shells during handling, and the density difference between shells and pipet tips allowed us to clearly separate these two materials.

Micro-CT scanning was consistently carried out for each of the specimens to obtain 3D renderings of the original shells (Table S4). All individual 3D renderings had a resolution of 1.45 µm and a voxel size of 1.45 × 1.45 × 1.45 µm^3^. Each scan contained 1440 individual X-ray radiographs per specimen, which were assembled using the software NRecon ver. 1.6.6.0 (SkyScan). Reconstructed files for each specimen were used to compute a 3D rendering in AVIZO 9.2 software, a program we used for shell visualization and quantification. First, shell material was segmented from the reconstructed radiographs by using a threshold of 11–13, an arbitrary value embedded in AVIZO to distinguish shell from non-shell material. Then, the embedded thickness-measuring tool ‘*Thickness map*’ in Avizo was used to calculate shell thickness (µm) along the complete surface in the binary image, defined as the diameter of the largest ball containing the voxels. This yielded more than 10,000,000 thickness estimates distributed over the entire surface area of each shell. Subsequently, shell thickness frequency distributions with a bin width of 2 µm were computed by the 3D software (see Fig. 3 for two examples), and average thickness per shell was calculated from these distributions. For eight specimens we calculated the empirical probability density distribution of normalized shell thickness by following these three steps: (1) first, we calculated the relative frequency distribution by dividing the number of thickness estimates within each bin by the total number of shell thickness estimates for the specimen concerned (Fig. S2a); (2) then, we normalized shell thickness by dividing the shell thickness data by the average shell thickness of the specimen concerned (x-axis in Fig. S2b); (3) finally, we calculated the empirical probability density distribution by dividing the relative frequency distribution by the normalized bin width, to correct for differences in bin width after the normalization step (y-axis in Fig. S2b). Diameter and height (µm) of the shells were measured using the simple measuring tool in AVIZO, and number of whorls was counted based on Kerney and Cameron’s method^52^ (Fig. S7).

Surface area dissolution was measured using a Field Emission Gun Scanning Electron Microscope (JSM7600F FEG SEM, JEOL, Benelux). Four of the 80 shells were broken after micro-CT scanning despite extreme care taken during handling, and were therefore not suitable for SEM analyses. Preparation of the 76 remaining specimens for SEM analyses was kept to a minimum to avoid damaging the fragile shells. We rinsed the shells three times using MilliQ water buffered with 10 mM NH_4_OH (pH 12) and dried shells for 24 h in a desiccator. After being placed on a stub in consistent orientation, specimens were coated with 10 nm gold–palladium in a sputter-coater. Dissolution was assessed based on each of the three recognized dissolution types^14^ (Fig. 4). The surface area (µm^2^) of each patch of dissolution was measured by selecting the dissolution marks on the 2D SEM images with the ‘magic wand’ option in Adobe Photoshop CS4.0 (Adobe Systems, San Jose, CA). The percentage surface area affected by dissolution was calculated for each shell, as the surface area on the first two whorls that is damaged by dissolution relative to the total surface area of the first two whorls (Fig. S8).

Light microscopy and SEM images of all investigated shells are provided in Fig. S9.

### Statistical analyses

Statistical analyses were conducted in R (R Core Team, 2018), using the packages *lme4*^53^ and vegan^54^. Relationships among the oceanographic variables (Ω_Ar_, pH, *p*CO_2_, temperature, oxygen and chlorophyll fluorescence) measured at the different stations were described by a Principal Component Analysis (PCA). To account for strong collinearity of several ocean variables, we conducted Principal Component Regression (PCR) with shell thickness as the dependent variable and the first two principal components obtained by the PCA as explanatory variables. An advantage of this regression approach is that, by definition, the principal components of a PCA are orthogonal and hence uncorrelated. For this analysis, we aggregated the average shell thicknesses of the multiple pteropods collected at each station into a single weighted mean shell thickness per station to avoid pseudo-replication.

To assess biometric relationships (shell thickness, diameter, height and number of whorls), Pearson’s product-moment correlation was used with a strict Bonferroni correction. To establish whether average shell thickness varied among stations, a one-way ANOVA was performed. The relationship between average shell thickness and the area of dissolution (%) was also assessed by Pearson’s product-moment correlation. Average shell thickness and the area of dissolution had an approximate normal distribution and did not deviate significantly from the assumption of homogeneity of variance across the stations (Levene’s test: p = 0.59 and p = 0.73, respectively).

### Genetic analyses

To assess genetic variability, mitochondrial cytochrome oxidase 1 (CO1) gene sequences were collected for 158 *L. helicina* specimens collected from the same stations (GenBank accession numbers MW022261-022417; Table S1). DNA was extracted from entire individuals using the protocol of Wall-Palmer et al.^55^. The nucleotide diversity (π) was determined in Arlequin v3.5.22^56^. To have an estimate of the genealogical relationships among *L. helicina* specimens, haplotype networks were constructed in Haploview^57^ based on Maximum Likelihood (ML) trees, which were generated in MEGA7^58^. ML trees were built upon best-fit models, estimated by jModelTest 2.0^59^. The outcome was an HKY model for *L. helicina* derived from the CCE, and a JC model for *L. helicina* from all regions (Fig. S6).

## Supplementary Information

Supplementary Information.

## Data Availability

Micro-CT scans are available from the corresponding author upon reasonable request. Mitochondrial cytochrome oxidase 1 (CO1) gene sequences have been deposited in BOLD and GenBank with accession numbers AAB8895 and MW022261-022417, respectively. All other data supporting the findings of this study are available within the paper and its supplementary information files.
