# Supplementary material for: Pteropods make thinner shells in the upwelling region of the California Current Ecosystem
Source: Sci Rep. 2021 Jan 18;11:1731. doi: 10.1038/s41598-021-81131-9 (PMC7814018; doi:10.1038/s41598-021-81131-9)
Supplement: Supplementary file 1 — Supplementary Information. [file 41598_2021_81131_MOESM1_ESM.pdf]

Supplementary Information

Pteropods make thinner shells in the upwelling region of the California Current Ecosystem

Lisette Mekkes[1,2*], Willem Renema[1], Nina Bednaršek[3], Simone R. Alin[4], Richard A. Feely[4], Jef Huisman[2], Peter Roessingh[5], Katja T. C. A. Peijnenburg[1,2*]

[1]Naturalis Biodiversity Center, Leiden, The Netherlands

[2]Department of Freshwater and Marine Ecology, Institute for Biodiversity and Ecosystem Dynamics, University of Amsterdam, Amsterdam, The Netherlands

[3]Southern California Coastal Water Research Project, Costa Mesa, California, USA

[4]Pacific Marine Environmental Laboratory, National Oceanic and Atmospheric Administration, Seattle, Washington, USA

[5]Department of Evolutionary and Population Biology, Institute for Biodiversity and Ecosystem Dynamics, University of Amsterdam, Amsterdam, The Netherlands

Supplementary Figures

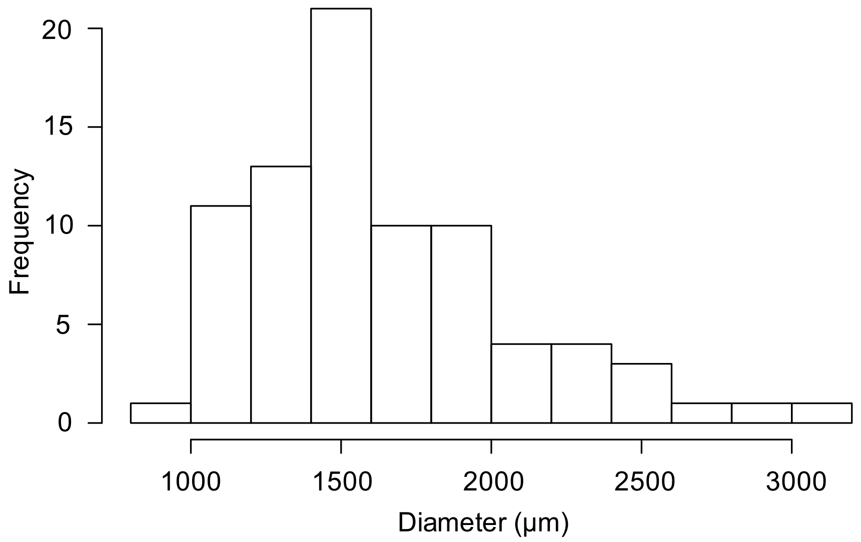

Fig. S1. Histogram of the frequency distribution of shell diameter among the analysed specimens of *Limacina helicina* from the California Current Ecosystem. Shell diameter had an approximate log-normal distribution and data did not deviate significantly from the assumption of homogeneity of variance (Levene's test over the log function: p=0.06).

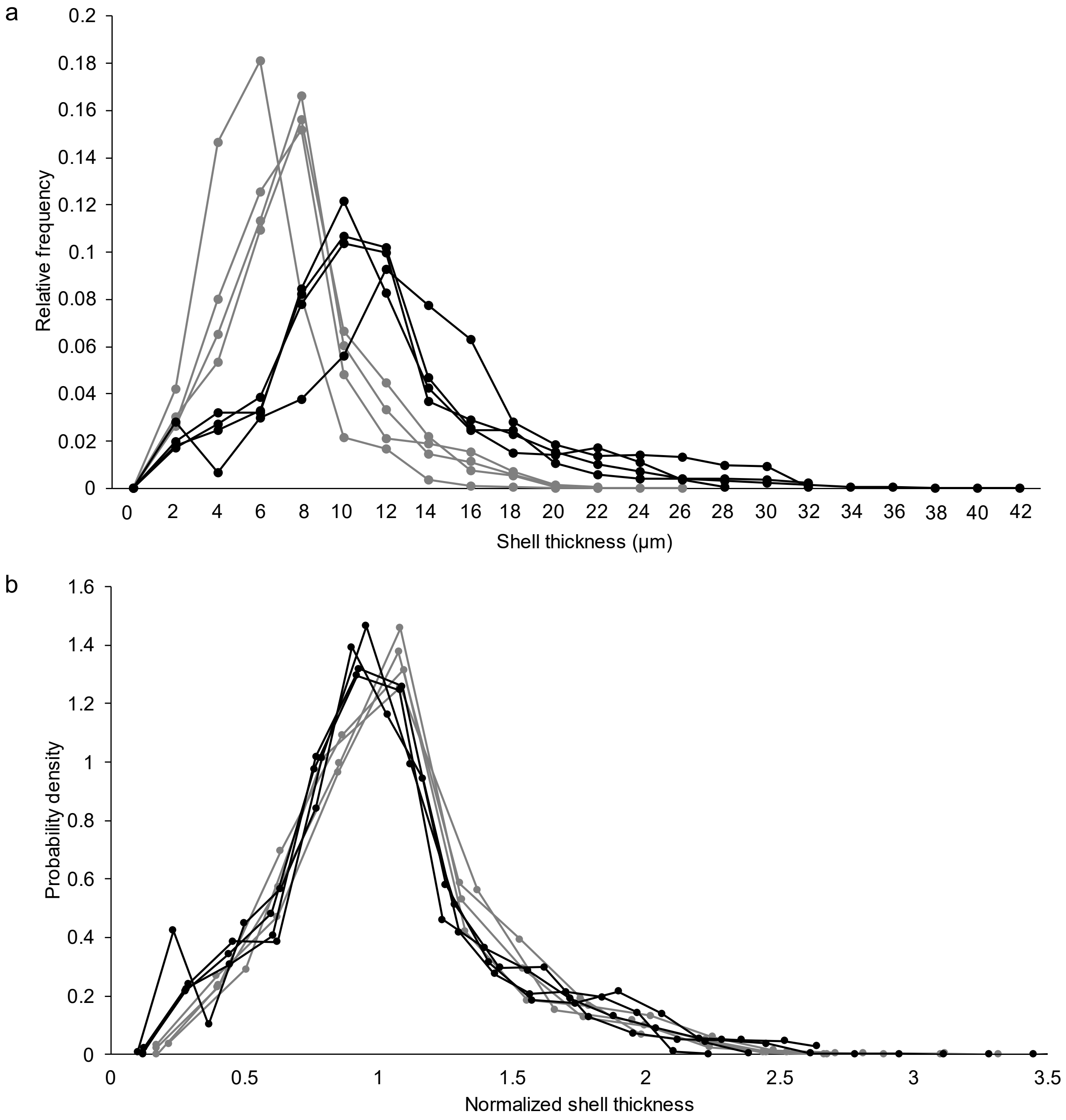

Fig. S2: The frequency distributions of shell thickness had a similar shape for all N=8 shells (same individuals as Fig. 4), indicating that shell thickening or thinning occurs more or less evenly across the entire shell. Each line represents the frequency distribution of shell thickness measurements of one individual shell; four thicker (black) and four thinner (grey) shells (~11-14 μm and ~6-8 μm average shell thickness, respectively). (**a**) Relative frequency distributions of shell thickness. The relative frequency distributions are similar in shape and are either located

more on the left side of the x-axis (thinner shells) or located more on the right side of the x-axis (thicker shells). (**b**) Probability density distributions of normalized shell thickness.

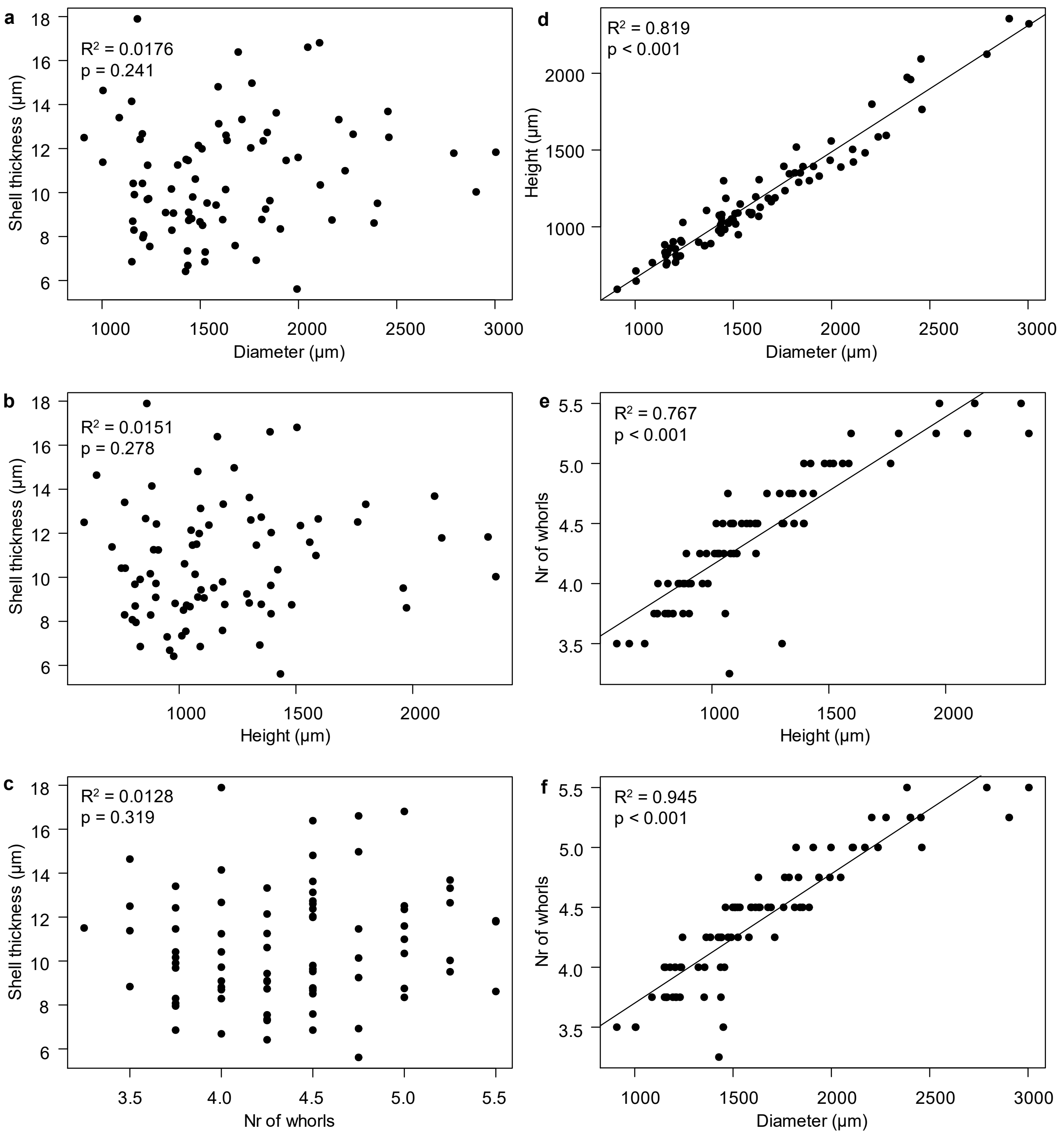

Fig. S3. Biometric correlations between shell parameters of *Limacina helicina*. Shell thickness is not correlated with (**a**) shell diameter, (**b**) shell height, or (**c)** number of whorls. Significant positive correlations were found among (**d)** shell diameter and height, (**e)** shell height and number of whorls, and (**f)** shell diameter and number of whorls (Pearson's product-moment correlations, all p Bonf. <0.01). The data consist of biometric measurements of *n*=80 individuals.

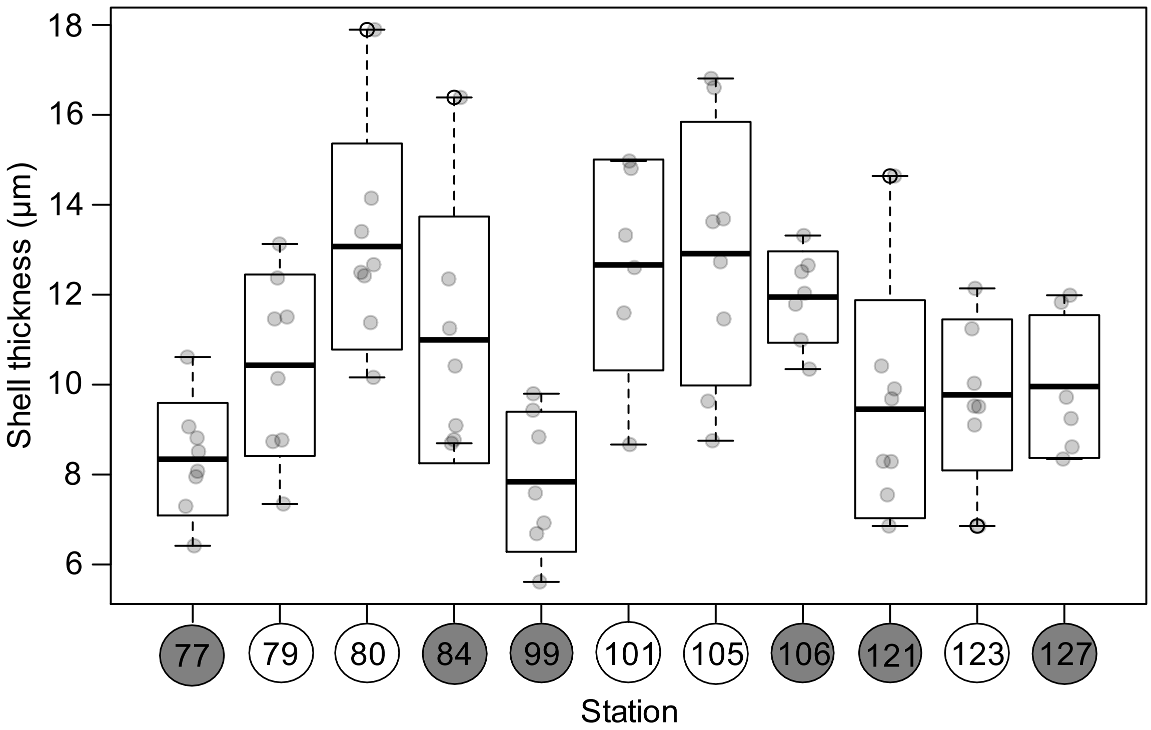

Fig. S4. Box plots of shell thickness of *Limacina helicina* at the different stations in the California Current Ecosystem (see Fig. 1). The horizontal line within the boxes represents the mean, the boxes are the standard deviations, and the bars are the minimum and maximum values. Scattered dots represent all individual measurements of shell thickness (six to eight individuals per station). Grey circles indicate nearshore stations, and white circles indicate offshore stations. Shell thickness varied significantly between the stations (one-way ANOVA: $F_{10,69} = 6.064$, $p<0.001$). Bars with different letters denote significant differences in mean shell thickness among stations (Tukey's HSD: $p<0.05$).

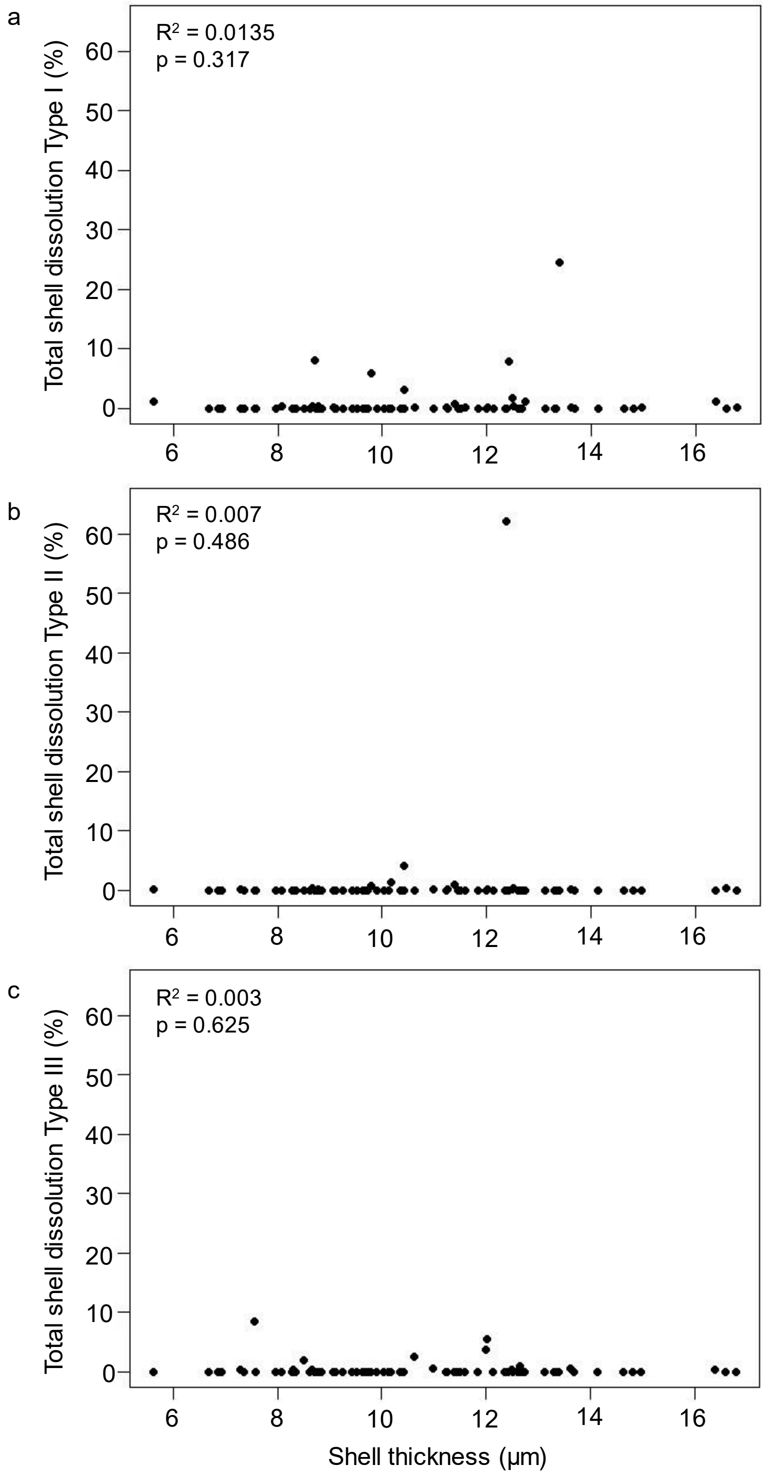

Fig. S5. Percentage dissolution (%) per dissolution type. a) Type I, b) Type II, and c) Type III. Each of the types was plotted against shell thickness and showed no correlation (Pearson's product-moment correlation r = 0.116, p = 0.317, r = 0.081, p = 0.486, and r = 0.0569, p = 0.625,

respectively). Each dot represents an individual pteropod analysed for both shell thickness and dissolution. Dissolution is expressed as percentage of the shell's surface area that is covered by dissolution marks (see Fig. S8 for an illustration of the measuring method and Fig. 4 for examples).

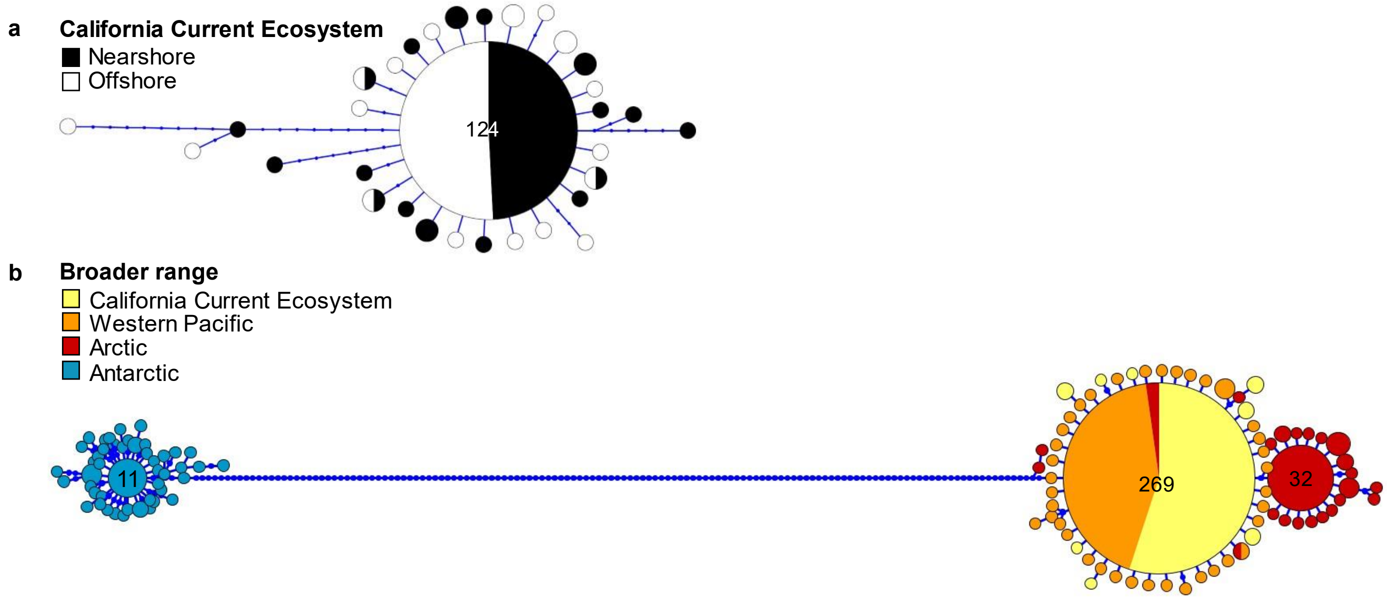

Fig. S6. Haplotype networks based on mitochondrial Cytochrome Oxidase I gene sequences of *Limacina helicina* individuals. Each circle is a unique haplotype, and the size of the circle is proportional to haplotype frequency (the numbers of individuals sharing the central haplotypes are indicated). Small blue dots represent the number of mutational steps between unique haplotypes. (**a**) Haplotype network for all *L. helicina* (nearshore and more offshore) from the California Current Ecosystem, indicating that a genetically homogeneous population was sampled in this study. (**b**) Haplotype network for *L. helicina* from different oceanographic regions: California Current Ecosystem (this study), western Pacific (*33*), Arctic (*34*,*35*) and Antarctic (*34*,*35*).

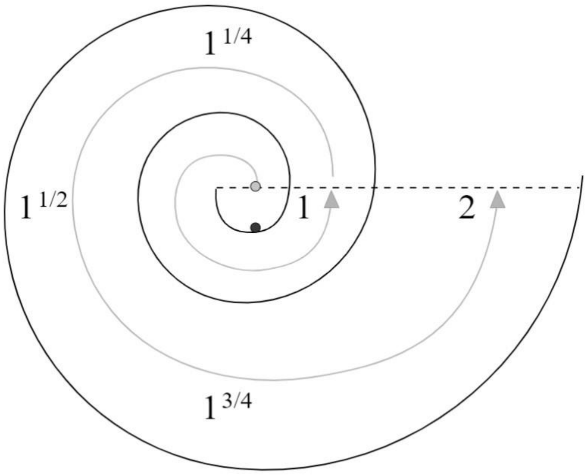

Fig. S7: Method of counting the number of whorls from a low-spired gastropod (*53*).

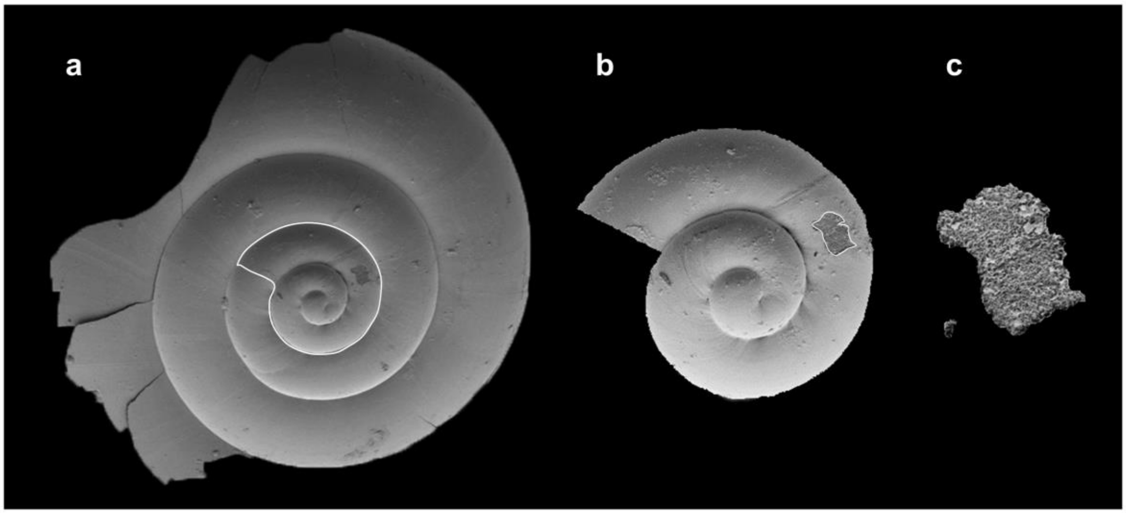

Fig. S8. Calculation of the percentage dissolution. (**a**) The entire pteropod shell, (**b**) selection of the first two whorls of the shell, based on Kerney & Cameron's (1979) (*53*) method, and (**c**) selection of marks of dissolution on the surface area located at the first two whorls. The percentage dissolution was calculated for each shell, as the surface area on the first two whorls that is covered by dissolution marks (**c**) relative to the total surface area of the first two whorls (**b**).

77-02
77-04
77-05
77-06
77-07
77-08

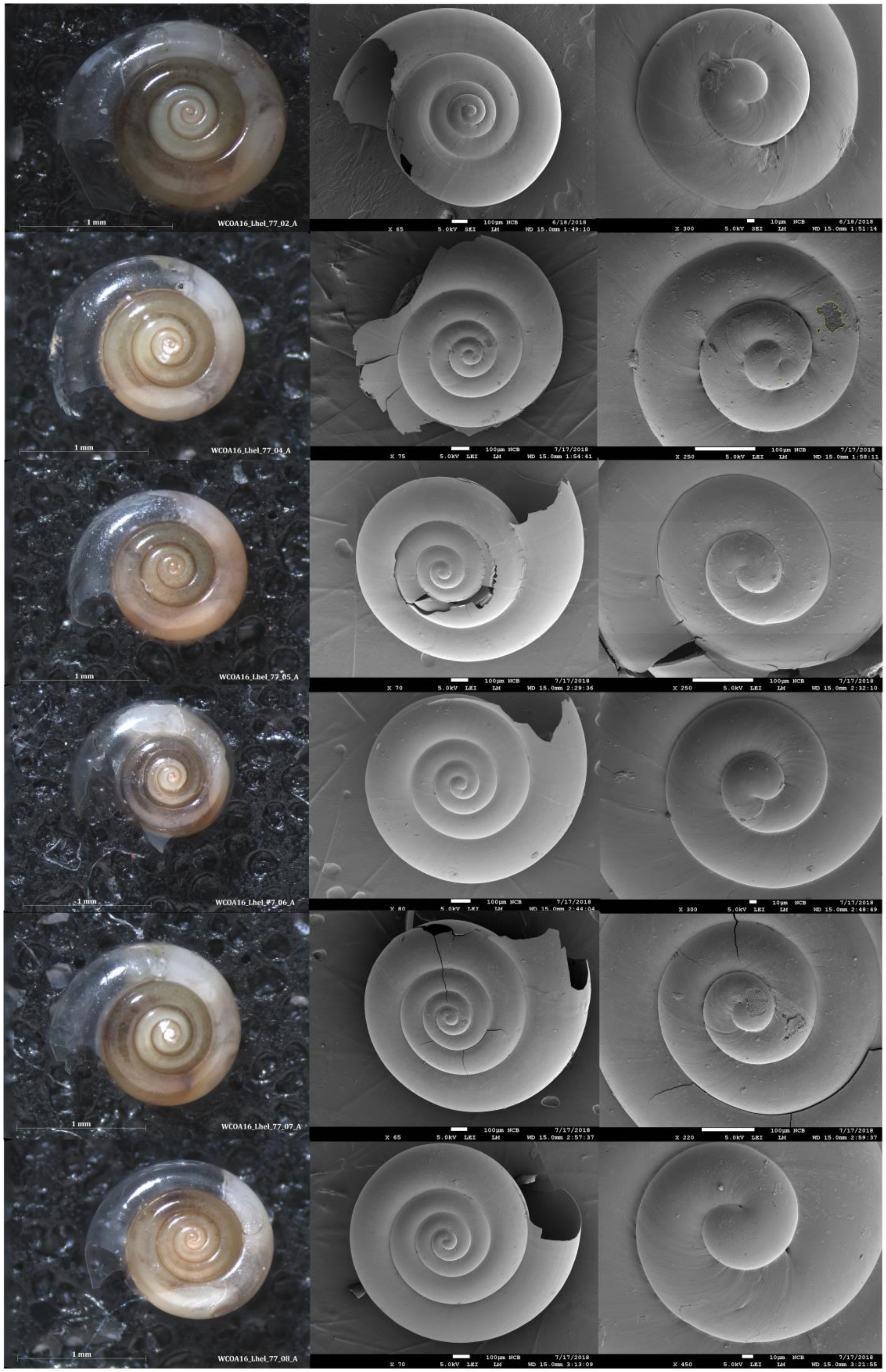

79-01

79-02

79-03

79-04

79-05

79-06

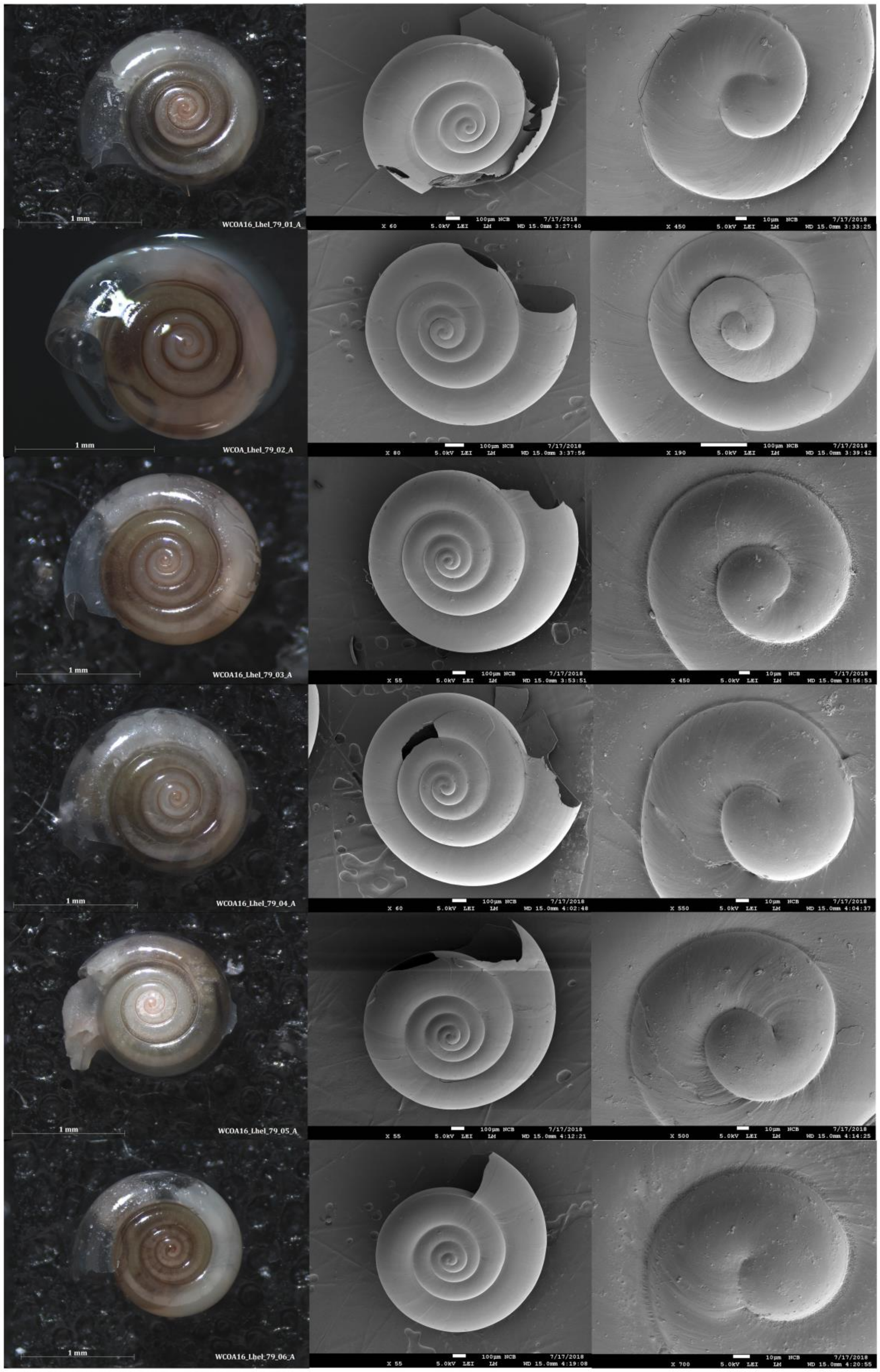

79-07

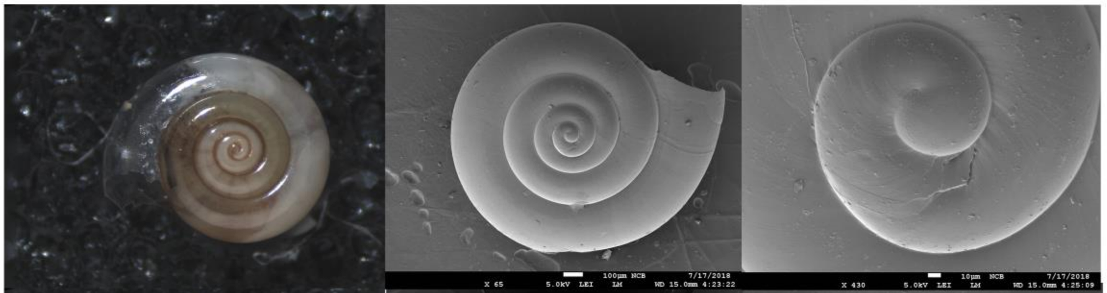

79-08

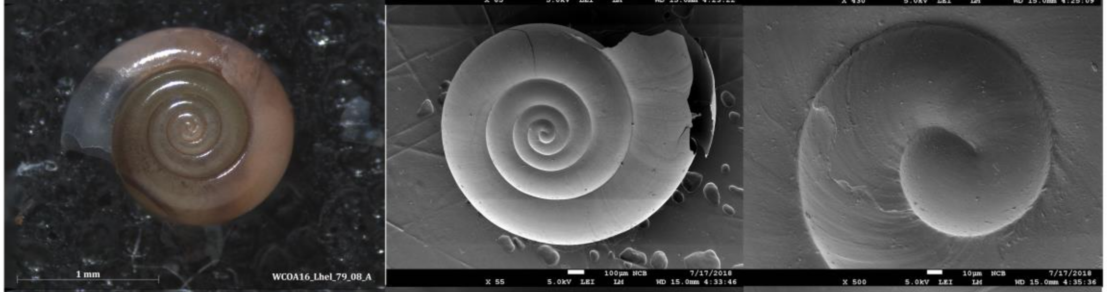

80-02

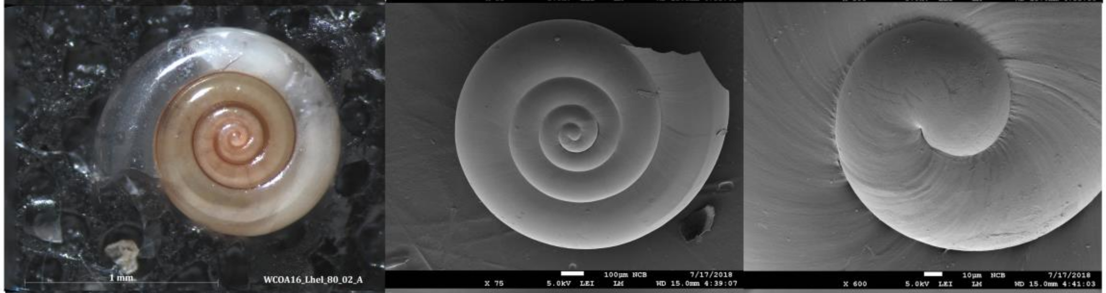

80-03

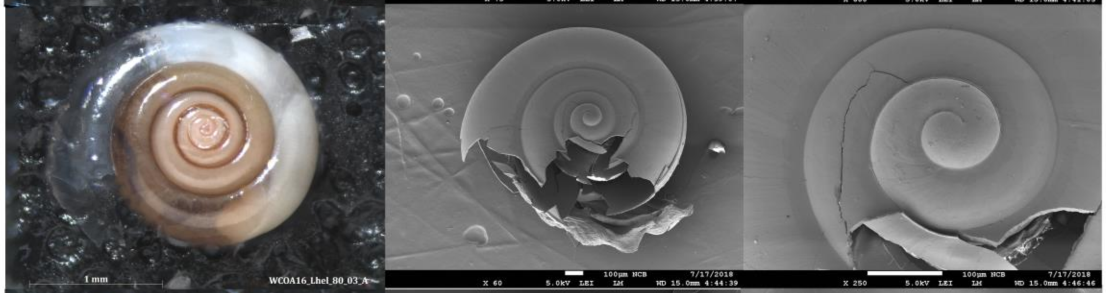

80-04

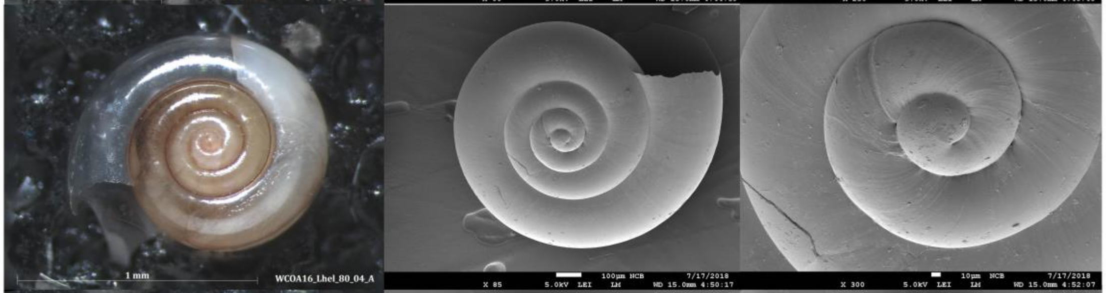

80-05

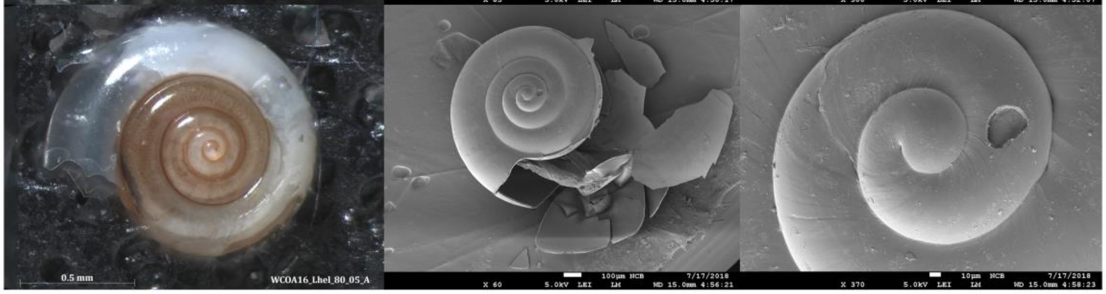

80-06
80-07
80-08
84-01
84-02
84-04

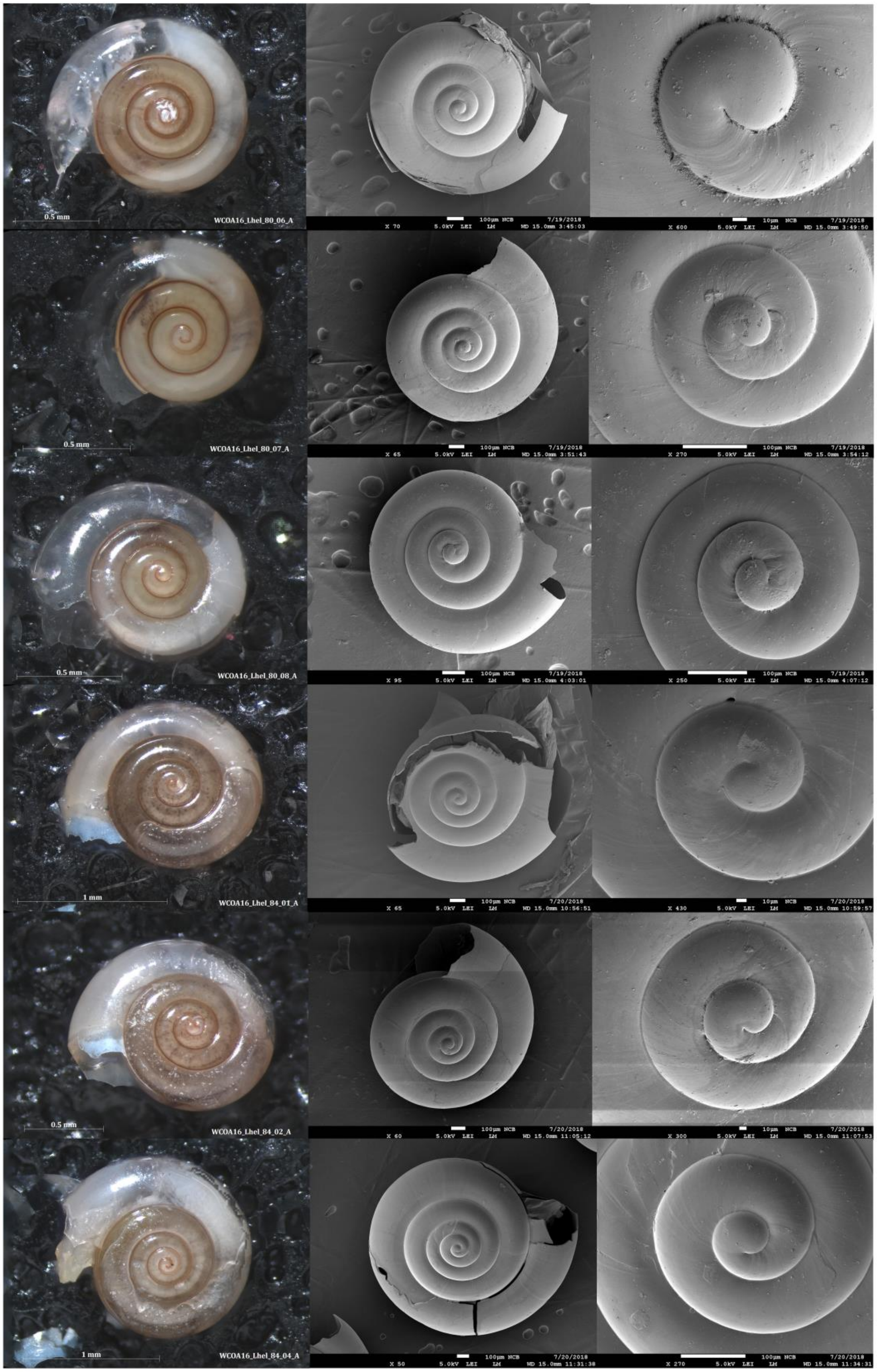

84-05
84-06
84-07
84-08
99-01
99-02

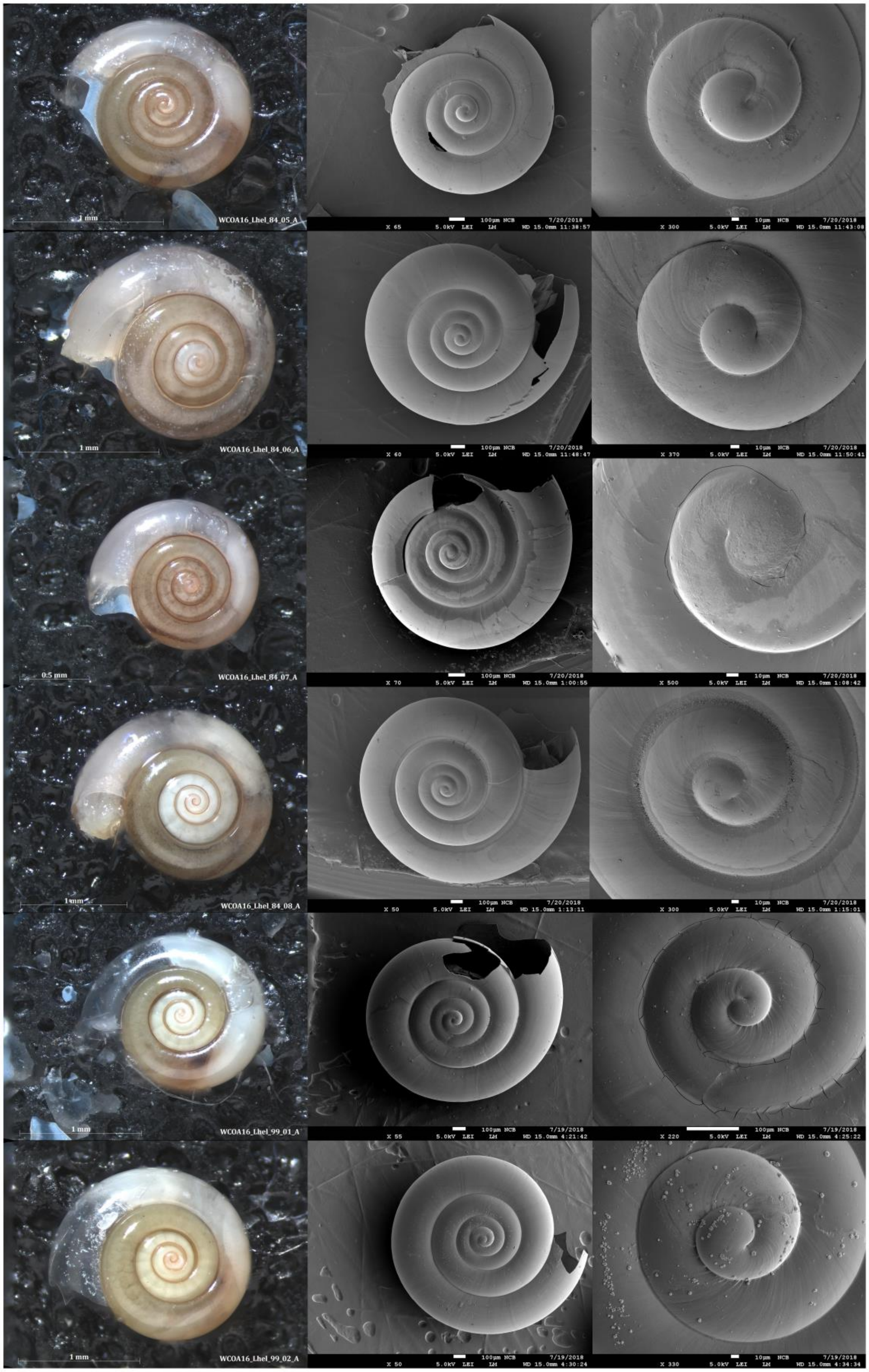

99-04
99-05
99-06
99-07
99-08
101-03

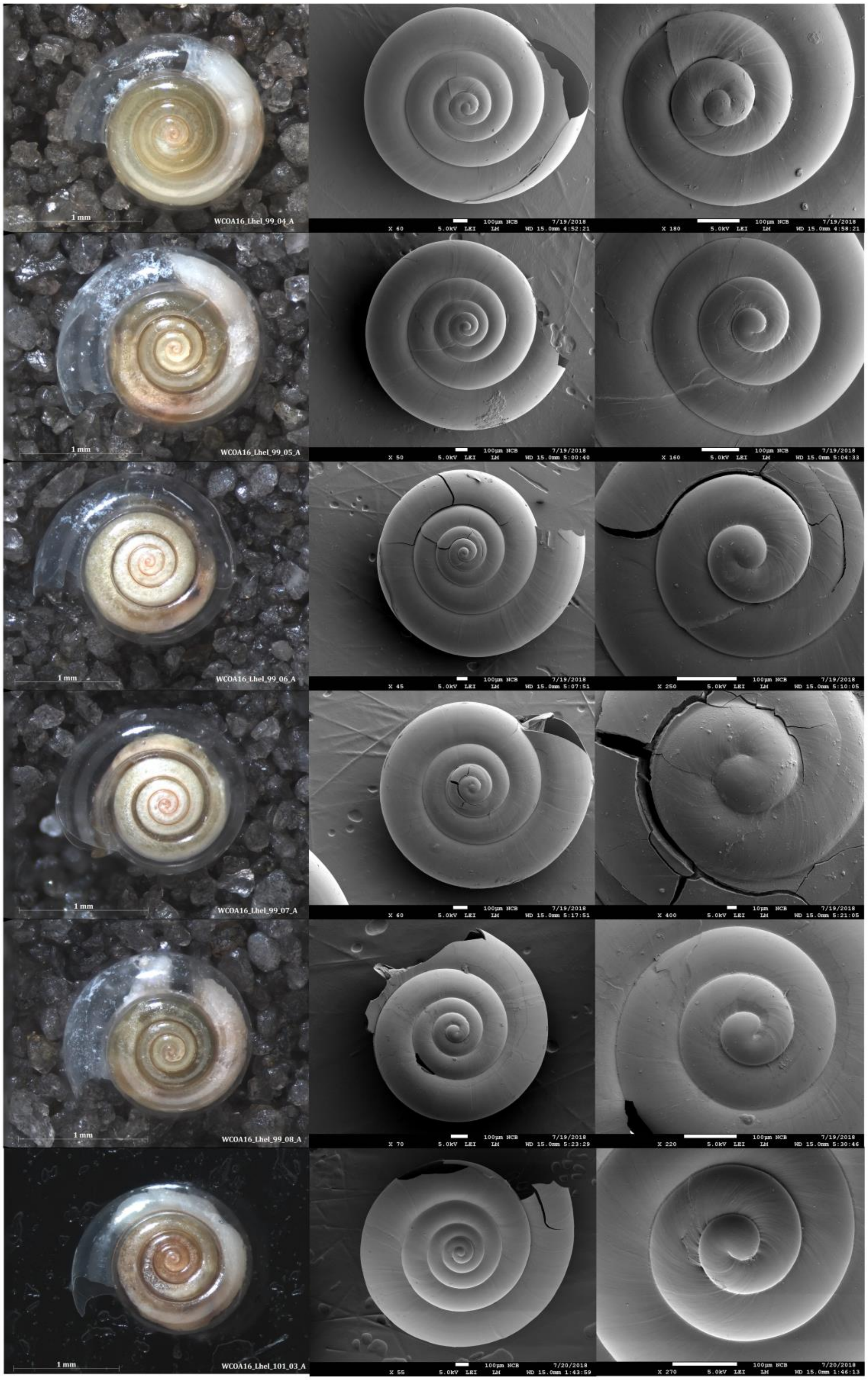

101-04

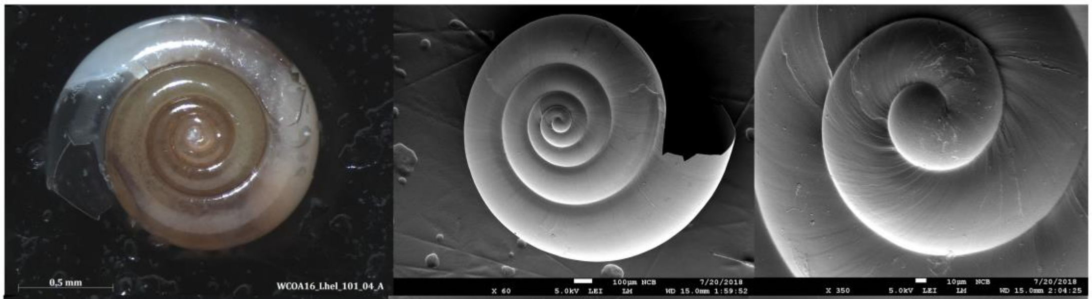

101-05

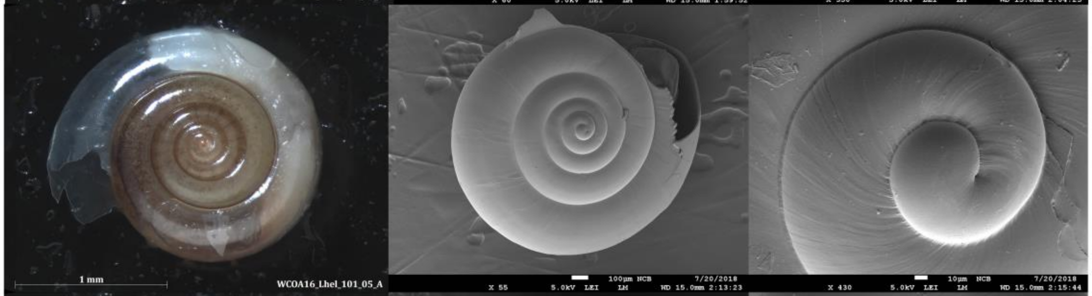

101-06

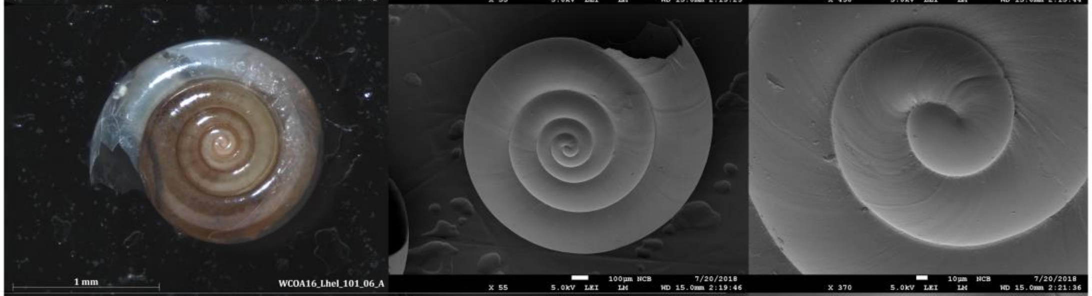

101-07

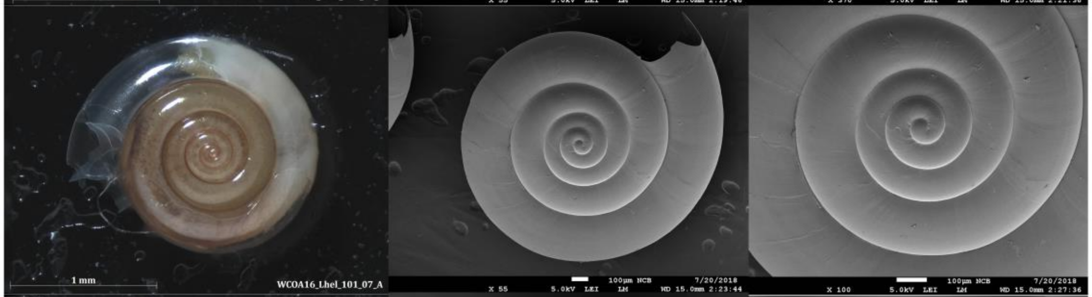

101-08

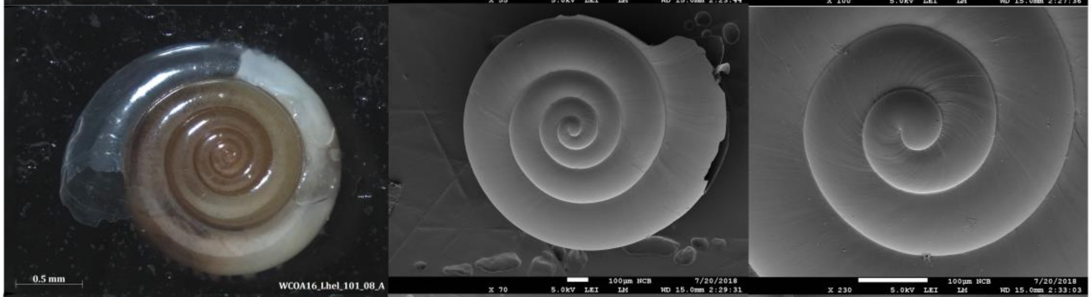

105-01

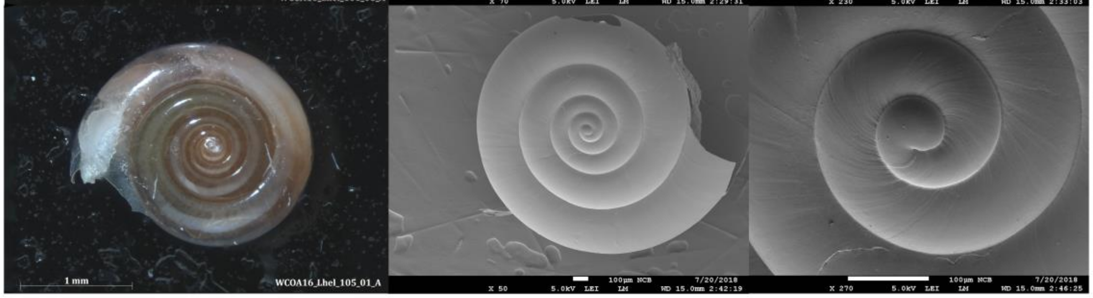

105-02

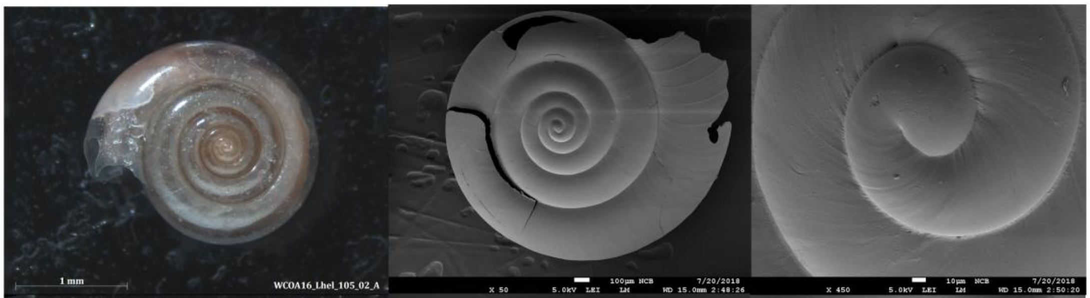

105-03

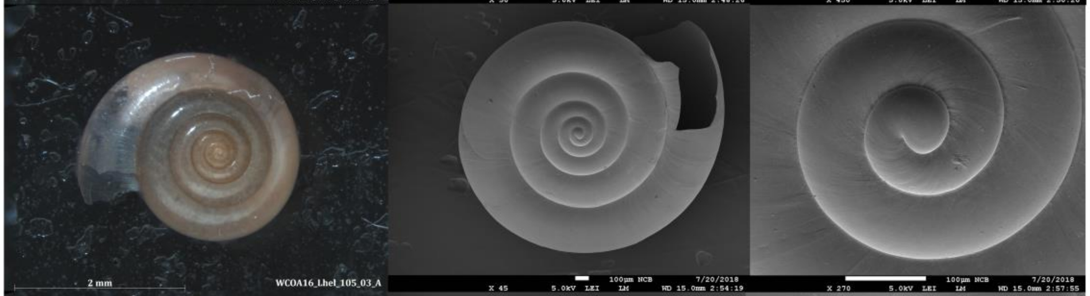

105-04

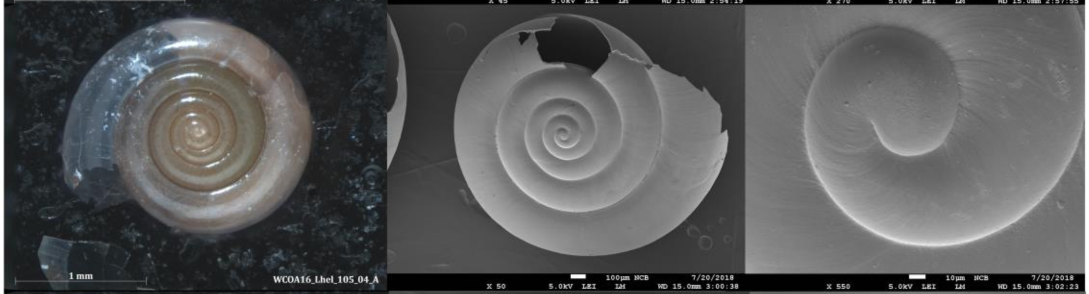

105-05

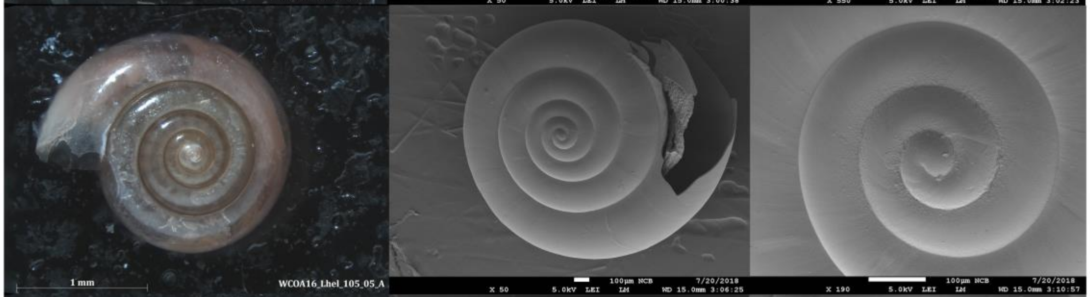

105-06

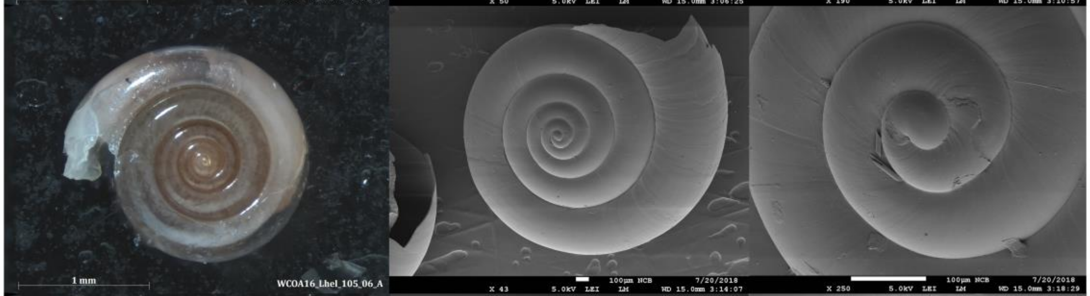

105-07

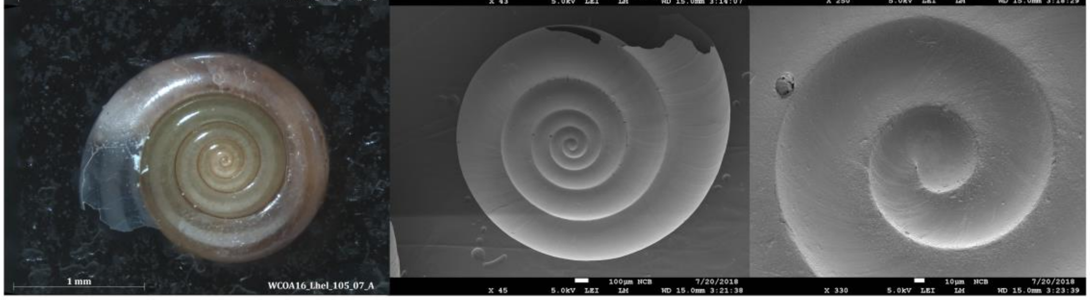

105-08

106-02

106-04

106-05

106-06

106-07

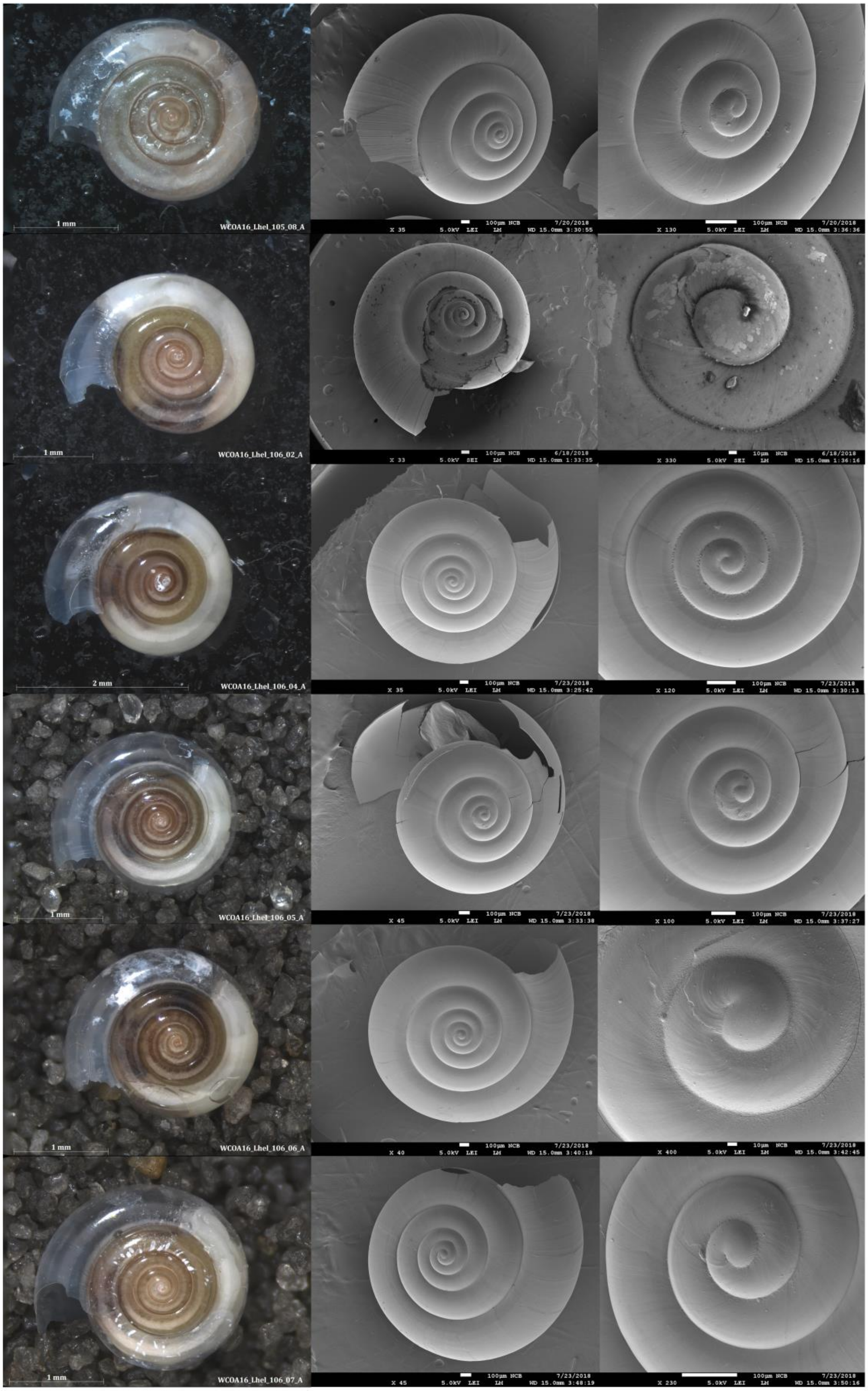

106-08

121-01

121-02

121-03

121-04

121-05

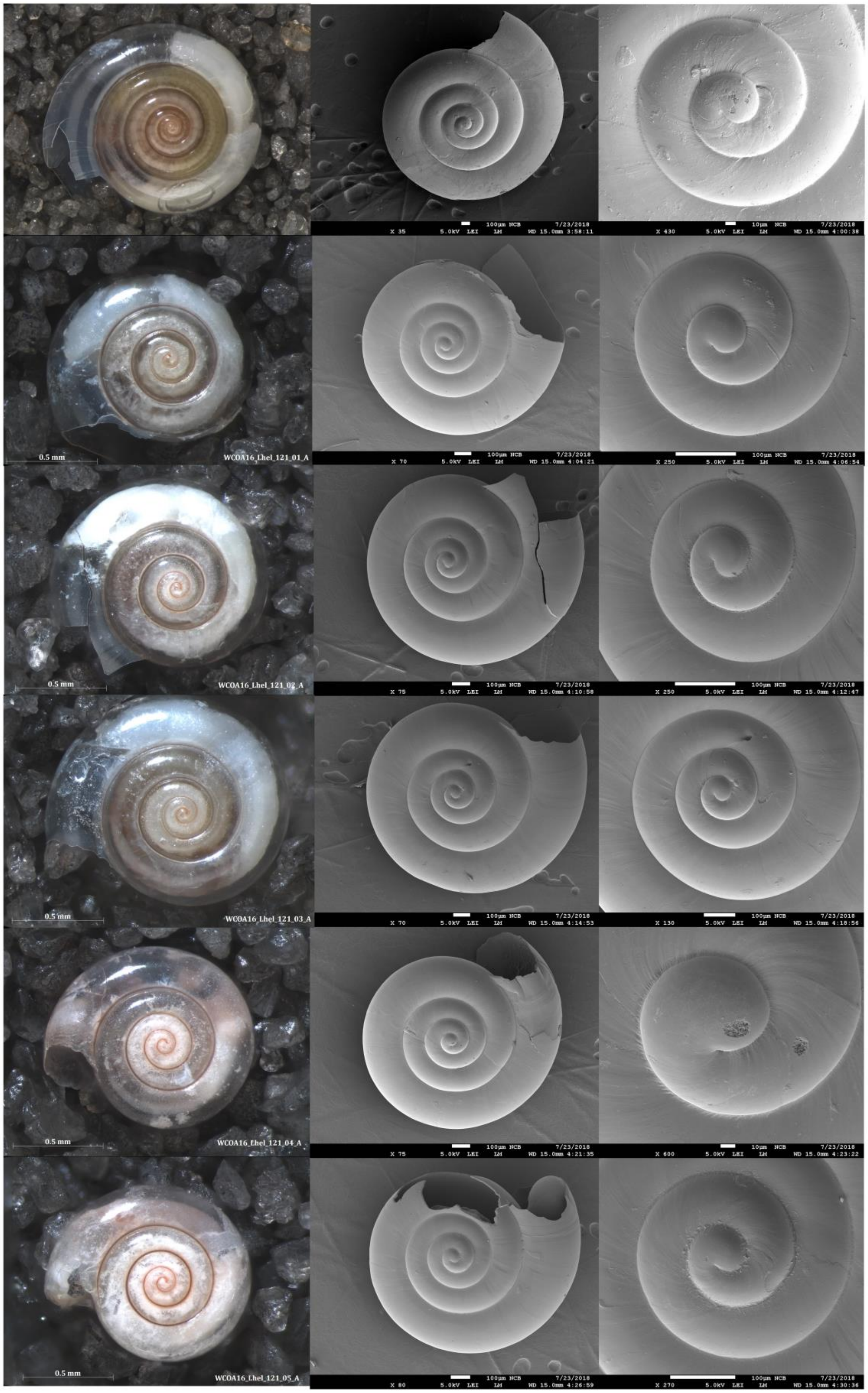

121-06

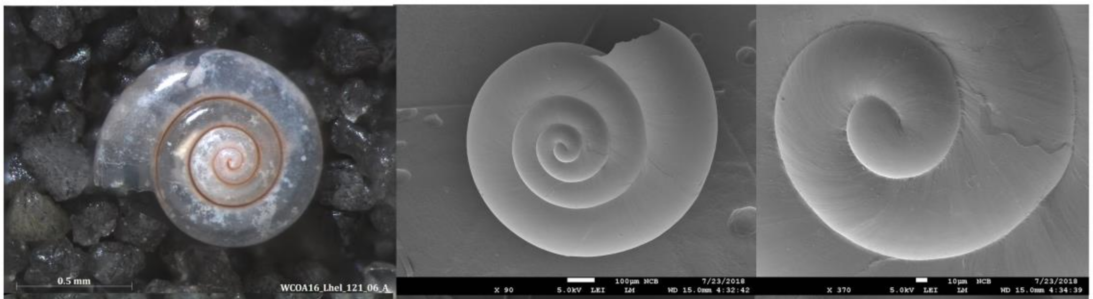

121-07

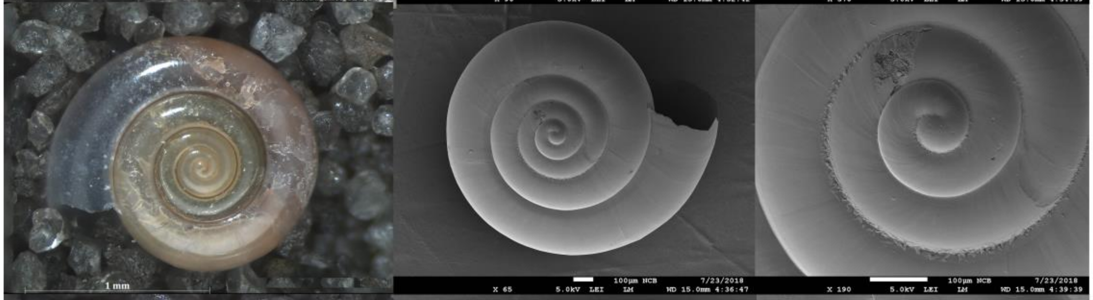

121-08

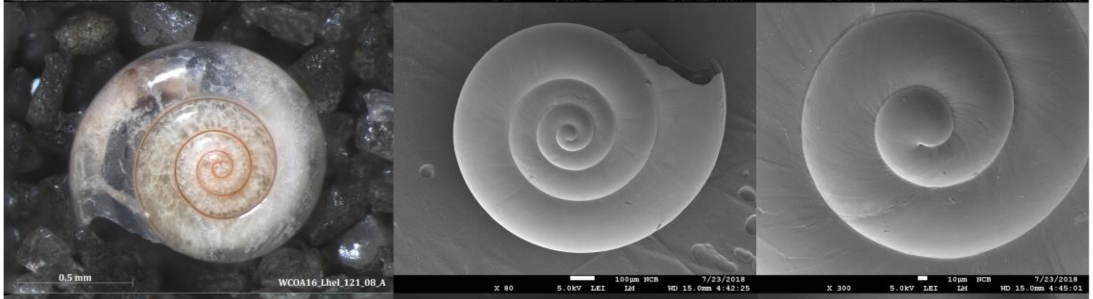

123-01

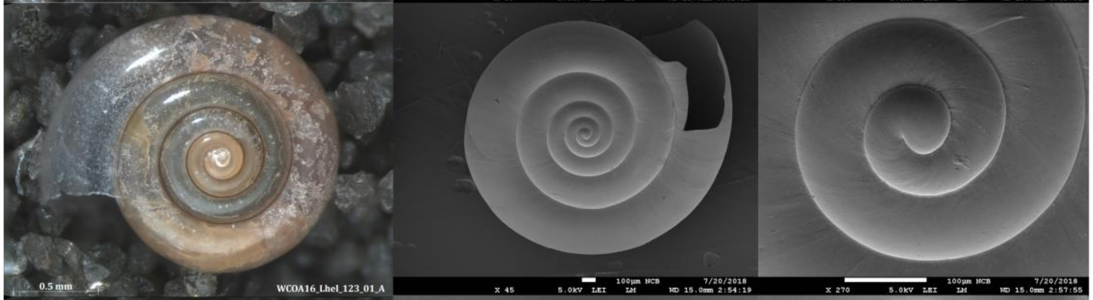

123-02

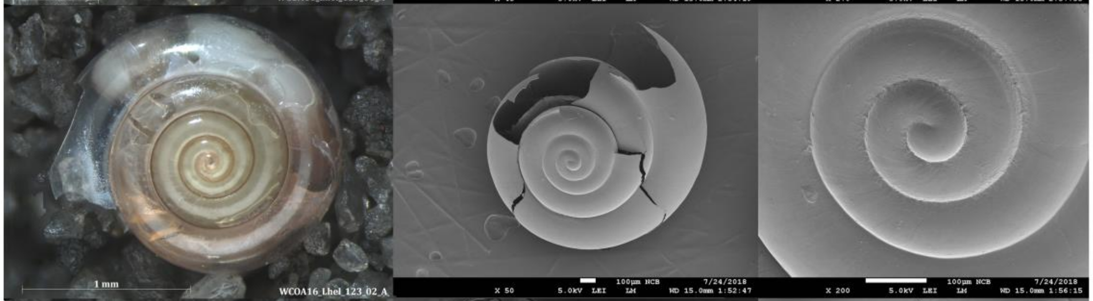

123-04

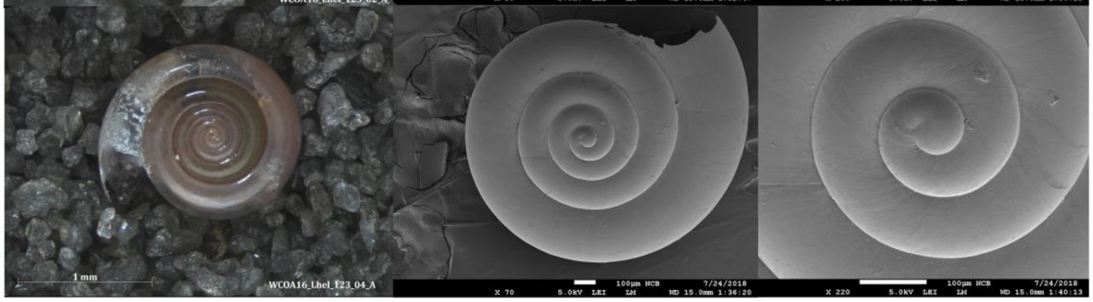

123-05

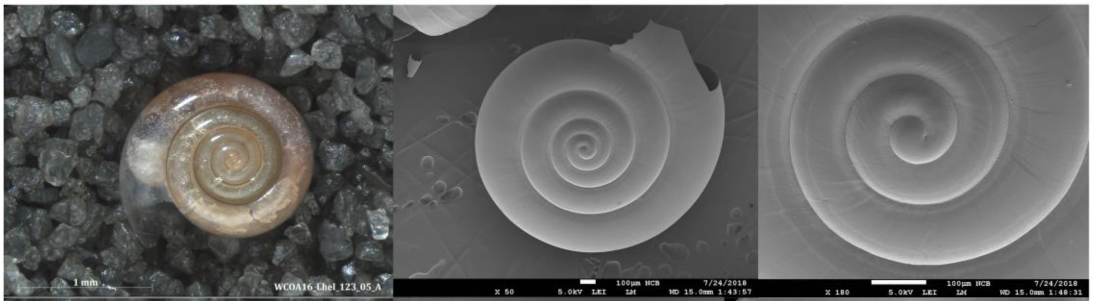

123-06

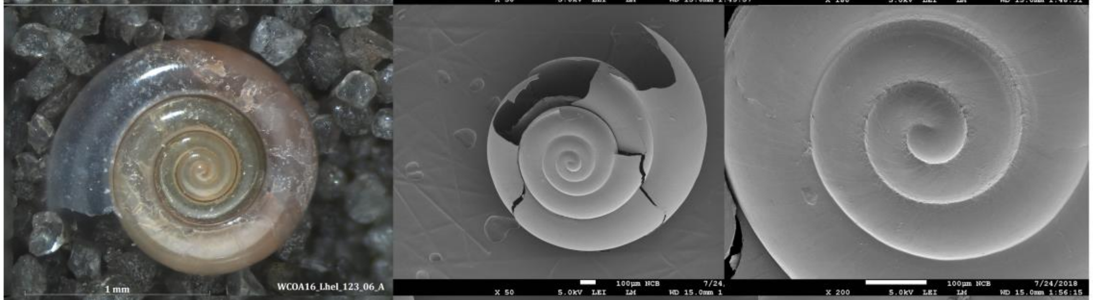

123-07

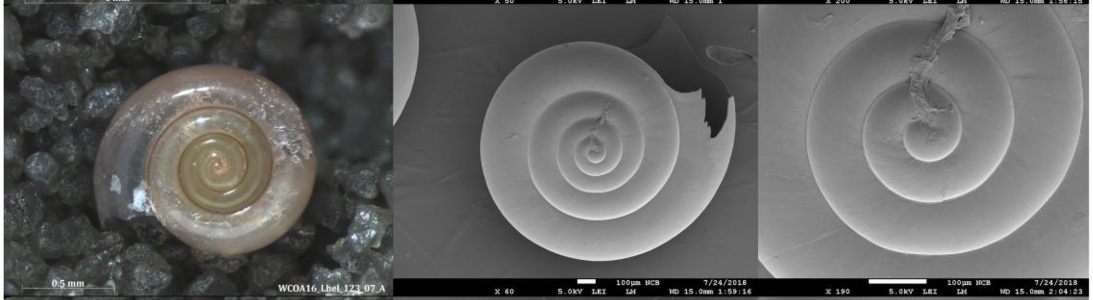

123-08

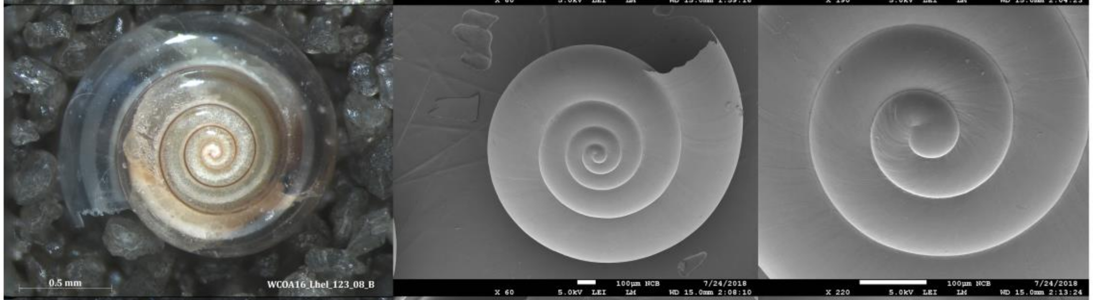

127-01

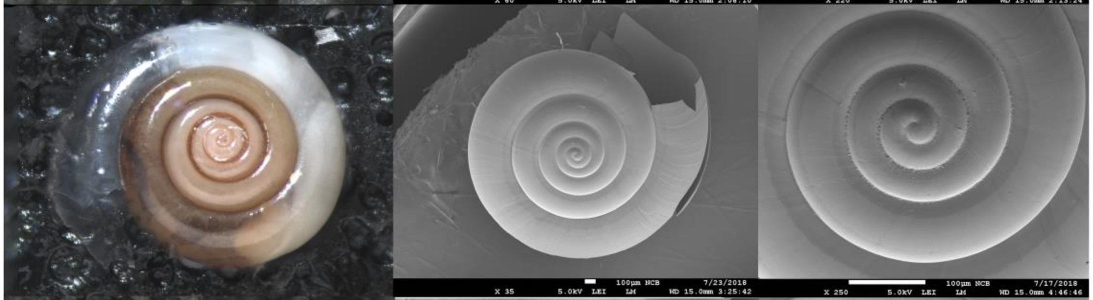

127-02

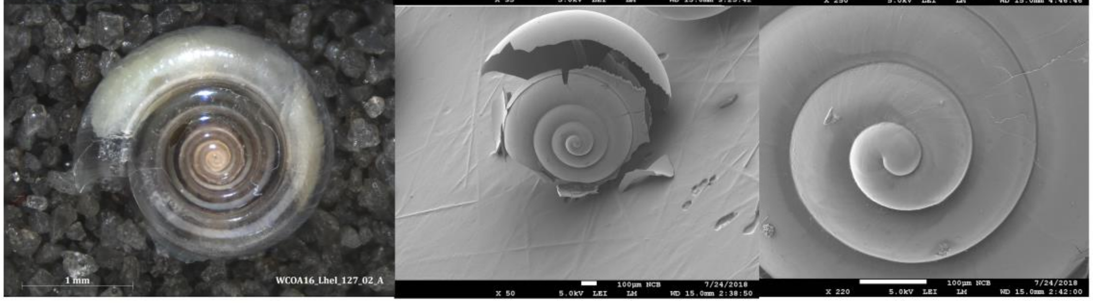

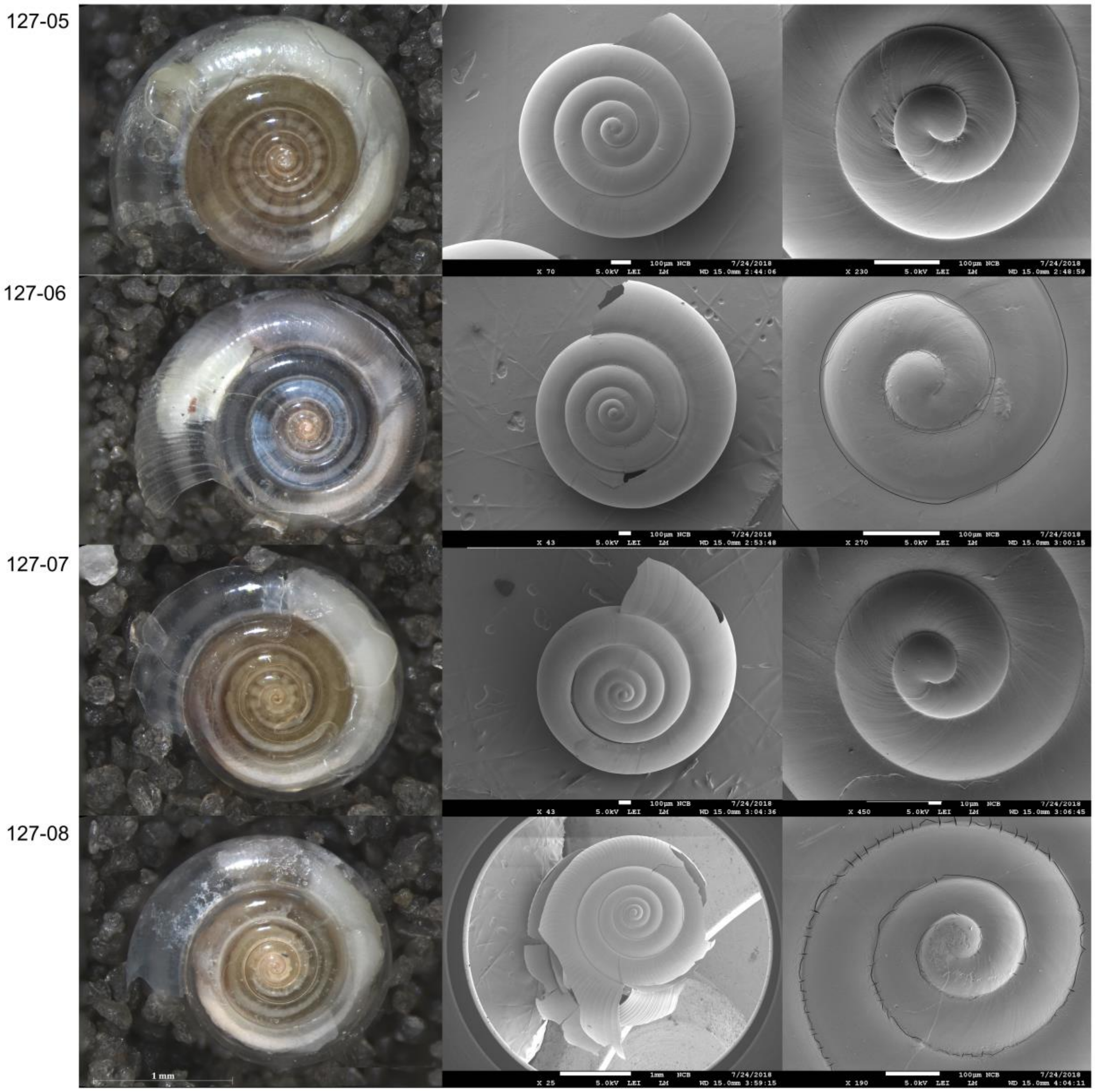

Fig. S9. Light microscope and SEM images of all analysed individuals of *Limacina helicina*.

Supplementary Tables

Table S1. Location, sampling dates, and number of *Limacina helicina* individuals used for analyses of shell thickness (Micro-CT), shell dissolution (SEM), and genetic variability (mitochondrial COI (mtCOI) barcoding). Mean shell thickness, mean shell diameter (± SD), and mean number of whorls of *L. helicina* at each station (based on *n*=6-8 individuals per station).

Station	Sampling date	Latitude	Longitude	No. of individuals			Shell thickness (μm)	Shell diameter (μm)	Number of whorls
				Micro-CT	SEM	mtCOI			
**77**	27 May 2016	44.2	-124.42	8	6	18	8.34 ± 1.25	1397.04 ± 125.87	4
**79**	28 May 2016	44.2	-124.98	8	8	20	10.43 ± 2.02	1526.93 ± 98.56	4
**80**	28 May 2016	44.65	-125.36	8	7	18	13.07 ± 2.29	1135.29 ± 135.65	4
**84**	29 May 2016	44.65	-124.65	7	7	18	10.99 ± 2.74	1485.33 ± 284.56	4
**99**	31 May 2016	47.12	-124.64	7	7	15	7.84 ± 1.56	1485.40 ± 0.02	4.5
**101**	31 May 2016	47.12	-124.98	6	6	16	12.66 ± 2.34	1626.24 ± 207.41	4.5
**105**	01 June 2016	47.68	-125.58	8	8	19	12.91 ± 2.93	2037.30 ± 207.74	4.5
**106**	01 June 2016	47.96	-125.33	7	6	15	11.94 ± 1.02	2262.69 ± 316.37	5
**121**	03 June 2016	48.49	-126.12	8	8	12	9.45 ± 2.42	1183.85 ± 99.70	4
**123**	03 June 2016	48.36	-126.30	7	7	0	9.77 ± 1.68	1789.63 ± 615.76	4.5
**127**	05 June 2016	51.83	-130.37	6	6	7	9.96 ± 1.59	1979.15 ± 634.65	4.5
			**Total**	**80**	**76**	**158**			

Table S2. All measurements per individual shell of *Limacina helicina*, used for analyses.

Station	Individual	Location	Nr whorls	Diameter (µm)	Height (µm)	Average shell thickness (µm)	Dissolution (μm^2) Type1	Dissolution (μm^2) Type2	Dissolution (μm^2) Type3	Shell surface (μm^2) inner two whorls
**77**	WCOA16_Lhel_77_02	Nearshore	4.25	1525.02	948.49	7.2952	0	124.52	334.87	69900.51
**77**	WCOA16_Lhel_77_04	Nearshore	4.5	1511.64	1018.45	8.51011	0	24.57	1412.97	73888.49
**77**	WCOA16_Lhel_77_05	Nearshore	3.75	1212	799.55	8.07365	328.57	0	0	70911.1
**77**	WCOA16_Lhel_77_06	Nearshore	3.75	1208.11	814.85	7.95147	0	0	0	-
**77**	WCOA16_Lhel_77_07	Nearshore	4.25	1474.78	1023	10.613	119.27	0	2164.11	85715.49
**77**	WCOA16_Lhel_77_08	Nearshore	4.25	1363.39	1106.55	9.06332	228.27	0	0	88788.18
**79**	WCOA16_Lhel_79_01	Offshore	3.75	1438.2	1057.15	11.4612	0	0	0	-
**79**	WCOA16_Lhel_79_02	Offshore	4.5	1636.42	1127.3	12.3723	0	51216.09	0	82494.74
**79**	WCOA16_Lhel_79_03	Offshore	4.75	1629.04	1068.15	10.1372	0	0	0	-
**79**	WCOA16_Lhel_79_04	Offshore	4.25	1434.72	1011.46	7.34532	34.19	0	0	87956.33
**79**	WCOA16_Lhel_79_05	Offshore	4.5	1593.86	1091.6	13.1277	0	0	0	-
**79**	WCOA16_Lhel_79_06	Offshore	3.25	1428.01	1074.68	11.5045	51.32	0	0	73802.32
**79**	WCOA16_Lhel_79_07	Offshore	4.25	1441.17	1031.7	8.73424	0	0	0	-
**79**	WCOA16_Lhel_79_08	Offshore	4.5	1614.01	1195.18	8.76762	45.32	0	0	78531.16
**80**	WCOA16_Lhel_80_02	Offshore	3.75	1193.71	902.57	12.4207	6287.78	0	0	79430.16
**80**	WCOA16_Lhel_80_03	Offshore	3.75	1353.05	876.35	10.1617	0	912.2	34.03	66919.413
**80**	WCOA16_Lhel_80_04	Offshore	3.75	1087.52	766.47	13.4044	0	20778.89	0	84976.07
**80**	WCOA16_Lhel_80_05	Offshore	4	1205.01	856.27	12.6681	23.21	0	0	74541.81
**80**	WCOA16_Lhel_80_06	Offshore	4	1150.73	882.92	14.1458	0	0	0	-
**80**	WCOA16_Lhel_80_07	Offshore	3.5	908.73	592.93	12.4976	1309.473	87.07	331.37	76536.64
**80**	WCOA16_Lhel_80_08	Offshore	3.5	1003.8	712.36	11.3798	576.98	827.23	0	82187.78
**84**	WCOA16_Lhel_84_01	Nearshore	4.5	1692.98	1163.48	16.387	1113.89	41.37	315.9	89387.71
**84**	WCOA16_Lhel_84_02	Nearshore	4	1323.45	899.54	9.08971	0	0	0	-
**84**	WCOA16_Lhel_84_04	Nearshore	4.25	1385.06	890.58	11.2518	0	86.34	0	88696.24
**84**	WCOA16_Lhel_84_05	Nearshore	5	1820.52	1519.26	12.3501	0	0	0	-
**84**	WCOA16_Lhel_84_06	Nearshore	4	1156.18	811.63	8.69619	6763.73	0	0	83406.37
**84**	WCOA16_Lhel_84_07	Nearshore	4	1205.75	768.92	10.4147	2371.72	3109.99	0	76373.23
**84**	WCOA16_Lhel_84_08	Nearshore	4.5	1813.35	1351.54	8.77476	243.49	158.71	0	84193.38

**99**	WCOA16_Lhel_99_01	Nearshore	4.25	1580.51	1093.39	9.43021	0	0	0	80857.19
**99**	WCOA16_Lhel_99_02	Nearshore	4.5	1677.47	1185.53	7.58811	0	0	0	-
**99**	WCOA16_Lhel_99_04	Nearshore	4.5	1461.45	1185.7	9.79744	5483.11	717.24	0	91775.79
**99**	WCOA16_Lhel_99_05	Nearshore	4.75	1784.83	1345.26	6.92417	0	0	0	-
**99**	WCOA16_Lhel_99_06	Nearshore	4.75	1992.52	1433.61	5.61277	1040.88	106.83	0	91212.02
**99**	WCOA16_Lhel_99_07	Nearshore	3.5	1450.21	1299.95	8.83584	0	0	0	-
**99**	WCOA16_Lhel_99_08	Nearshore	4	1436.66	959.37	6.68661	0	0	0	-
**101**	WCOA16_Lhel_101_03	Offshore	4.75	1762.79	1235.47	14.9729	110.77	0	0	65479.31
**101**	WCOA16_Lhel_101_04	Offshore	4.5	1498.46	1046.27	8.66775	209.83	282.42	229.13	71027.47
**101**	WCOA16_Lhel_101_05	Offshore	4.5	1630.95	1306.82	12.6045	20.19	0	0	67458.57
**101**	WCOA16_Lhel_101_06	Offshore	4.5	1590.79	1079.68	14.8085	0	0	0	-
**101**	WCOA16_Lhel_101_07	Offshore	4.25	1711.89	1188.59	13.3232	0	0	0	-
**101**	WCOA16_Lhel_101_08	Offshore	5	1998.08	1559.12	11.5945	78.72	0	0	84627.19
**105**	WCOA16_Lhel_105_01	Offshore	5	2170.39	1481.78	8.75191	0	0	0	-
**105**	WCOA16_Lhel_105_02	Offshore	5	2107.12	1504.38	16.8085	78.58	51.38	47.89	75449.25
**105**	WCOA16_Lhel_105_03	Offshore	5.25	2455.07	2093.62	13.6867	0	0	0	-
**105**	WCOA16_Lhel_105_04	Offshore	4.5	1840.83	1351.42	12.7325	856.53	0	0	76875
**105**	WCOA16_Lhel_105_05	Offshore	4.5	1854.22	1392.23	9.6323	56.48	0	0	83182.79
**105**	WCOA16_Lhel_105_06	Offshore	4.5	1886.69	1300.63	13.6247	247.47	85.99	478.05	86960.07
**105**	WCOA16_Lhel_105_07	Offshore	4.75	1936.94	1330.57	11.4612	418.56	0	0	79683.79
**105**	WCOA16_Lhel_105_08	Offshore	4.75	2047.16	1389.16	16.6071	0	269.13	0	65170.14
**106**	WCOA16_Lhel_106_02	Nearshore	5	2460	1764.54	12.512	269.03	239.01	36.96	76363.81
**106**	WCOA16_Lhel_106_04	Nearshore	5.25	2205.19	1798.93	13.3158	0	0	0	-
**106**	WCOA16_Lhel_106_05	Nearshore	4.5	1756.96	1393.96	12.0305	106.87	177.61	5312.06	97383.23
**106**	WCOA16_Lhel_106_06	Nearshore	5.25	2278.03	1595.37	12.6516	78.53	0	945.41	89418.74
**106**	WCOA16_Lhel_106_07	Nearshore	5	2110.88	1422.15	10.3452	0	0	0	-
**106**	WCOA16_Lhel_106_08	Nearshore	5	2237.24	1585.57	10.9901	0	5401.66	14269.02	2358362.19
**121**	WCOA16_Lhel_121_01	Nearshore	3.75	1164.55	832.84	9.90584	0	0	0	-
**121**	WCOA16_Lhel_121_02	Nearshore	3.75	1230.34	810.3	9.68329	0	0	0	-
**121**	WCOA16_Lhel_121_03	Nearshore	4	1355.35	877.93	8.28839	0	0	0	-
**121**	WCOA16_Lhel_121_04	Nearshore	3.75	1162.35	766.32	8.29529	0	209.57	866.48	296292.49
**121**	WCOA16_Lhel_121_05	Nearshore	3.75	1158.55	752.58	10.4149	32.77	0	26.44	77256.59

**121**	WCOA16_Lhel_121_06	Nearshore	3.5	1004.92	646.21	14.6398	0	0	0	-
**121**	WCOA16_Lhel_121_07	Nearshore	4.25	1242.95	1028.42	7.54926	8.58	0	6974.81	81864.95
**121**	WCOA16_Lhel_121_08	Nearshore	3.75	1151.78	833.45	6.85561	10.98	0	0	79894.42
**123**	WCOA16_Lhel_123_01	Offshore	5.25	2904.19	2355.91	10.0285	0	0	0	-
**123**	WCOA16_Lhel_123_02	Offshore	5.25	2401.85	1959.39	9.5108	15.66	70.23	0	83275.05
**123**	WCOA16_Lhel_123_04	Offshore	4.5	1534.62	1148.37	9.52406	12.37	0	0	89232.95
**123**	WCOA16_Lhel_123_05	Offshore	4.25	1440.89	1079.92	9.10314	0	0	0	-
**123**	WCOA16_Lhel_123_06	Offshore	4.25	1490.55	1051.05	12.1407	0	0	0	-
**123**	WCOA16_Lhel_123_07	Offshore	4	1232.07	910.57	11.2419	207.35	0	0	99445.69
**123**	WCOA16_Lhel_123_08	Offshore	4.5	1523.26	1090.17	6.85561	0	0	0	-
**127**	WCOA16_Lhel_127_01	Offshore	5.5	3004.38	2322.56	11.8329	0	0	0	-
**127**	WCOA16_Lhel_127_02	Offshore	5.5	2384.42	1973.39	8.6147	0	0	0	-
**127**	WCOA16_Lhel_127_05	Offshore	4.75	1832.7	1289.91	9.2456	43.23	0	0	81963.52
**127**	WCOA16_Lhel_127_06	Offshore	5	1907.63	1393.41	8.34506	0	0	0	-
**127**	WCOA16_Lhel_127_07	Offshore	4	1237.12	900.57	9.72042	0	0	0	-
**127**	WCOA16_Lhel_127_08	Offshore	4.5	1508.63	1085.92	11.9879	0	0	3388.61	91641.1

Table S3. Ocean data including temperature (°C), salinity (S), Dissolved Inorganic Carbon (DIC), Total Alkalinity (TA), silicate (Si) and phosphate (PO_4). The data represent measurements for each of the 11 stations for the upper 100 m of the water column. For two stations (coastal stations 77 and 99) the maximum depth was lower than 100 m.

**Station**	**Lat**	**Long**	**Pressure**	**Temp (°C)**	**S**	**DIC µmol/kg**	**TA µmol/kg**	**Si µmol/kg**	**PO_4 µmol/kg**
**77**	44.20	-124.42	3.28	11.51	31.82	1964.00	2168.20	4.12	0.41
			9.96	10.50	32.26	2022.20	2178.70	8.13	0.82
			20.14	9.38	32.64	2095.10	2187.70	19.13	1.49
			30.18	9.36	32.91	2105.30	2200.70	17.93	1.44
			39.96	8.96	33.32	2156.50	2225.10	25.53	1.81
			49.89	8.49	33.66	2195.70	2240.40	28.73	2.02
			59.80	8.26	33.73	2209.80	2247.60	34.94	2.15
			79.93	7.60	33.90	2239.80	2263.90	43.54	2.41
			90.00	7.59	33.90	2244.90	2266.10	45.34	2.40
**79**	44.20	-124.98	2.96	14.41	29.13	1828.00	2068.20	6.16	0.09
			10.44	12.55	31.70	1892.90	2110.70	4.96	0.18
			20.22	11.65	32.23	1990.90	2173.10	1.78	0.40
			30.35	10.63	32.36	2017.10	2176.60	3.57	0.60
			40.06	10.08	32.43	2032.90	2176.10	4.76	0.82
			49.76	9.94	32.46	2037.50	2176.90	5.55	0.86
			59.56	9.66	32.70	2080.30	2194.20	12.50	1.20
			80.26	9.12	33.29	2146.60	2222.30	23.20	1.68
			100.57	8.49	33.62	2186.20	2242.00	29.34	2.00
**80**	44.65	-125.36	4.04	15.27	30.08	1880.40	2099.50	0.94	0.09
			10.66	14.05	30.94	1941.90	2146.40	1.53	0.26
			20.85	11.98	32.29	1982.70	2172.90	2.12	0.39
			30.70	11.67	32.37	1987.70	2175.70	1.92	0.43
			40.30	10.83	32.46	2004.30	2176.10	2.70	0.54

			50.30	10.52	32.49	2016.80	2178.30	4.07	0.70
			60.38	10.20	32.54	2024.30	2177.20	5.25	0.79
			80.40	9.96	32.77	2063.90	2192.90	11.34	1.16
			100.42	9.22	33.32	2138.40	2218.60	22.52	1.72
**84**	44.65	-124.65	3.42	14.53	29.91	1838.70	2097.40	4.97	0.08
			10.54	14.50	29.91	1853.60	2104.30	5.58	0.13
			20.48	11.06	31.99	2002.20	2169.60	5.99	0.63
			30.53	10.27	32.41	2020.40	2174.80	3.85	0.73
			40.75	9.99	32.49	2026.40	2174.70	4.85	0.83
			50.03	9.90	32.66	2059.20	2187.60	10.36	1.11
			59.85	9.43	32.94	2104.80	2205.10	17.64	1.52
			80.66	8.91	33.37	2160.60	2230.00	26.09	1.90
			100.19	8.45	33.62	2189.50	2244.30	32.58	2.15
**99**	47.12	-124.64	2.83	12.08	31.29	1844.90	2154.70	7.29	0.27
			9.93	12.03	31.30	1888.10	2155.50	8.77	0.33
			14.97	10.67	31.39	2022.90	2159.00	25.28	1.38
			19.89	9.99	31.67	2056.10	2163.30	26.26	1.64
			29.93	9.80	32.30	2050.50	2171.80	13.46	1.39
			40.05	9.25	32.61	2069.00	2186.70	15.43	1.28
			49.84	8.87	32.90	2124.50	2208.10	23.80	1.73
			60.03	8.69	33.30	2137.30	2219.90	22.33	1.71
			79.92	7.79	33.74	2230.40	2261.00	46.19	2.34
			88.81	7.68	33.80	2229.10	2261.10	46.44	2.32
**101**	47.12	-124.98	3.59	13.46	31.46	1906.20	2149.00	0.57	0.19
			10.56	13.46	31.46	1904.90	2147.50	0.58	0.20
			20.22	13.21	31.88	1958.40	2160.30	0.82	0.33
			30.52	11.24	32.19	1988.10	2169.90	2.77	0.52
			40.40	10.04	32.31	2010.70	2172.20	4.72	0.75
			50.40	9.74	32.44	2023.80	2178.40	5.94	0.87
			60.23	9.33	32.57	2054.30	2188.60	10.11	1.13

			80.19	8.87	33.30	2143.00	2225.40	22.86	1.74
			100.74	8.24	33.58	2167.50	2241.60	27.77	1.90
**105**	47.68	-125.58	3.18	13.73	31.80	1952.00	2153.40	1.23	0.32
			5.06	13.73	31.80	1951.40	2153.40	1.23	0.32
			10.11	13.73	31.80	1951.40	2153.30	1.24	0.32
			19.84	13.62	31.79	1953.00	2137.00	1.24	0.33
			29.94	12.16	31.90	1963.30	2158.50	2.07	0.40
			40.36	10.35	32.34	2008.70	2172.90	3.93	0.69
			49.86	9.82	32.43	2020.80	2176.70	5.37	0.82
			59.90	9.40	32.52	2033.90	2181.40	7.43	0.95
			79.70	9.25	32.87	2082.60	2198.00	14.44	1.32
			99.80	8.73	33.29	2134.60	2221.90	22.88	1.71
**106**	47.96	-125.33	2.97	13.47	31.68	1932.10	2153.70	1.43	0.25
			10.29	12.05	32.01	1952.10	2159.30	4.92	0.35
			20.48	10.55	32.13	2001.10	2163.70	6.36	0.69
			30.45	9.61	32.43	2034.10	2174.50	7.17	0.91
			40.78	9.31	32.58	2050.20	2183.30	10.04	1.04
			50.67	9.14	32.74	2070.00	2181.00	12.71	1.17
			61.07	8.90	32.97	2102.20	2205.10	17.83	1.41
			80.83	8.53	33.30	2136.60	2222.00	23.98	1.67
			100.57	8.18	33.55	2160.40	2238.70	28.28	1.82
**121**	48.49	-126.12	2.77	13.01	31.88	1946.50	2157.80	0.88	0.31
			5.18	13.01	31.88	1947.80	2157.70	0.87	0.31
			10.44	12.34	32.00	1956.00	2162.70	1.85	0.34
			19.80	10.63	31.99	1985.80	2160.00	6.20	0.66
			29.87	9.76	32.07	2020.70	2163.00	10.55	0.96
			40.19	8.91	32.47	2052.70	2177.90	14.89	1.03
			50.33	8.73	32.75	2073.90	2193.20	16.06	1.25
			60.34	8.66	32.94	2098.30	2203.40	19.02	1.42
			80.12	8.18	33.39	2149.30	2227.10	27.90	1.75

			100.15	7.99	33.66	2169.20	2244.60	30.26	1.84
**123**	48.36	-126.31	3.41	13.04	31.72	1940.80	2149.70	1.89	0.35
			9.56	13.01	31.72	1940.10	2149.80	1.88	0.34
			20.57	11.87	32.01	1962.30	2160.00	4.05	0.40
			31.11	10.12	32.14	2013.30	2165.50	6.80	0.80
			39.47	9.48	32.31	2034.70	2170.70	9.95	0.97
			50.38	9.24	32.45	2041.90	2175.60	10.73	1.02
			60.32	9.07	32.67	2063.20	2188.90	12.10	1.12
			79.92	8.93	32.96	2094.20	2204.80	16.43	1.34
			100.68	8.51	33.41	2145.90	2226.40	24.49	1.72
**127**	51.83	-130.37	3.08	12.61	31.30	1918.40	2120.60	2.29	0.38
			10.24	12.26	31.31	1920.40	2121.30	2.48	0.37
			19.62	11.81	31.36	1932.00	2121.40	3.27	0.44
			29.60	10.61	31.64	1965.80	2135.40	6.02	0.65
			39.90	10.29	31.65	1978.70	2134.00	7.99	0.72
			59.26	9.51	31.92	2004.80	2149.70	11.33	0.90
			79.72	9.21	32.18	2024.40	2159.00	12.71	0.99
			98.63	8.84	32.49	2054.60	2179.60	15.85	1.11

Table S4. Settings for Micro-CT scanning and X-ray alignment in NRecon to generate 3D renderings in Avizo 9.0 3D software (FEI, 2007).

**Micro-CT**	Current	168 µA
	Voltage	60 kV
	Power	10 W
	Rotation step	0.25°
	Resolution	high
	Image pixel size	1.45 µm
	Camera binning	2 x 2
	Target material (source)	Tungsten
	Tube	Sealed microfocus X-ray tube, air cooled
	Detector	11 Megapixel (4000 × 2300) 12-bit digital CCD-camera
	Exposure (ms)	1160
	Beam hardening correction	0.6
**NRecon**	Reconstruction angular range	360°
	Result type file	.bmp
